# Ecological divergence and conservatism: spatiotemporal patterns of niche evolution in a genus of livebearing fishes (Poeciliidae: *Xiphophorus*)

**DOI:** 10.1186/s12862-016-0593-4

**Published:** 2016-02-19

**Authors:** Zachary W. Culumber, Michael Tobler

**Affiliations:** Division of Biology, Kansas State University, Manhattan, KS 66506 USA

**Keywords:** Phyloclimatic modeling, Geographic distribution, Disparity, Thermal biology

## Abstract

**Background:**

Ecological factors often have a strong impact on spatiotemporal patterns of biodiversity. The integration of spatial ecology and phylogenetics allows for rigorous tests of whether speciation is associated with niche conservatism (constraints on ecological divergence) or niche divergence. We address this question in a genus of livebearing fishes for which the role of sexual selection in speciation has long been studied, but in which the potential role of ecological divergence during speciation has not been tested.

**Results:**

By combining reconstruction of ancestral climate tolerances and disparity indices, we show that the earliest evolutionary split in *Xiphophorus* was associated with significant divergence for temperature variables. Niche evolution and present day niches were most closely associated with each species’ geographic distribution relative to a biogeographic barrier, the Trans-Mexican Volcanic Belt. Tests for similarity of the environmental backgrounds of closely related species suggested that the relative importance of niche conservatism and divergence during speciation varied among the primary clades of *Xiphophorus*. Closely related species in the two swordtail clades exhibited higher levels of niche overlap than expected given environmental background similarity indicative of niche conservatism. In contrast, almost all species of platyfish had significantly divergent niches compared to environmental backgrounds, which is indicative of niche divergence.

**Conclusion:**

The results suggest that the relative importance of niche conservatism and divergence differed among the clades of *Xiphophorus* and that traits associated with niche evolution may be more evolutionarily labile in the platyfishes. Our results ultimately suggest that the taxonomic scale of tests for conservatism and divergence could greatly influence inferences of their relative importance in the speciation process.

**Electronic supplementary material:**

The online version of this article (doi:10.1186/s12862-016-0593-4) contains supplementary material, which is available to authorized users.

## Background

Ecological factors play a critical role in evolutionary processes by providing sources of selection that drive microevolutionary change and by imposing constraints that limit organismal performance [1–3]. Accordingly, they can shape phenotypic evolution and functional diversification and set the distributional limits for populations and species [4–6]. Ecological factors can also directly drive speciation. Ecological speciation models suggest that divergent natural selection drives the emergence of reproductive isolation as a byproduct of adaptation [7, 8]. Speciation in this context is accompanied by divergence in niche occupation along at least one axis of multidimensional niche space [9, 10]. Even when non-ecological factors drive the initial emergence of reproductive isolation, multifarious selection can stabilize and accelerate speciation processes [11] and lead to the secondary accumulation of ecological differences among lineages [12–15]. Alternatively, constraints imposed by ecological factors can also play a role in speciation, if evolutionary lineages become geographically isolated during periods of environmental change but retain ancestral niche affinities (niche conservatism [16]). In this case, vicariant speciation occurs when ancestral distributional ranges shift and break up (e.g., along elevational gradients during periods of warming or orogeny), and incipient species fail to adapt to novel environmental conditions that would facilitate the maintenance of gene flow [16, 17]. The relative contributions of niche divergence and niche conservatism during speciation processes should consequently affect the functional diversification of lineages and shape the degree of niche similarity between members of a phylogenetic group. However, the relative importance of these factors during diversification remains largely unknown. The integration of ecological modeling and biogeographic and phylogenetic analyses allows for comparative analyses of niche evolution across broad spatial scales, which can provide insight into the relationship between ecological differentiation and diversification across large taxonomic groups [18–20].

The fish family Poeciliidae has risen as a prominent model for investigating phenotypic evolution and speciation in response to sexual selection [21, 22]. Accordingly, we have a fairly sophisticated understanding of how female preferences have shaped the evolution of sexually selected traits [23–27]. There is also an emerging understanding of how natural selection mediated by physiochemical stressors and biotic interactions can drive adaptation and speciation in select groups (e.g., [28–31]). Nonetheless, the importance of ecological divergence at broader taxonomic scales and the relative contributions of ecological and sexual selection in speciation remain poorly understood [32]. Here we focus on one of the most diverse poeciliid genera to conduct the first broad comparative study of ecological niche divergence in livebearing fishes.

**Fig. 1:**
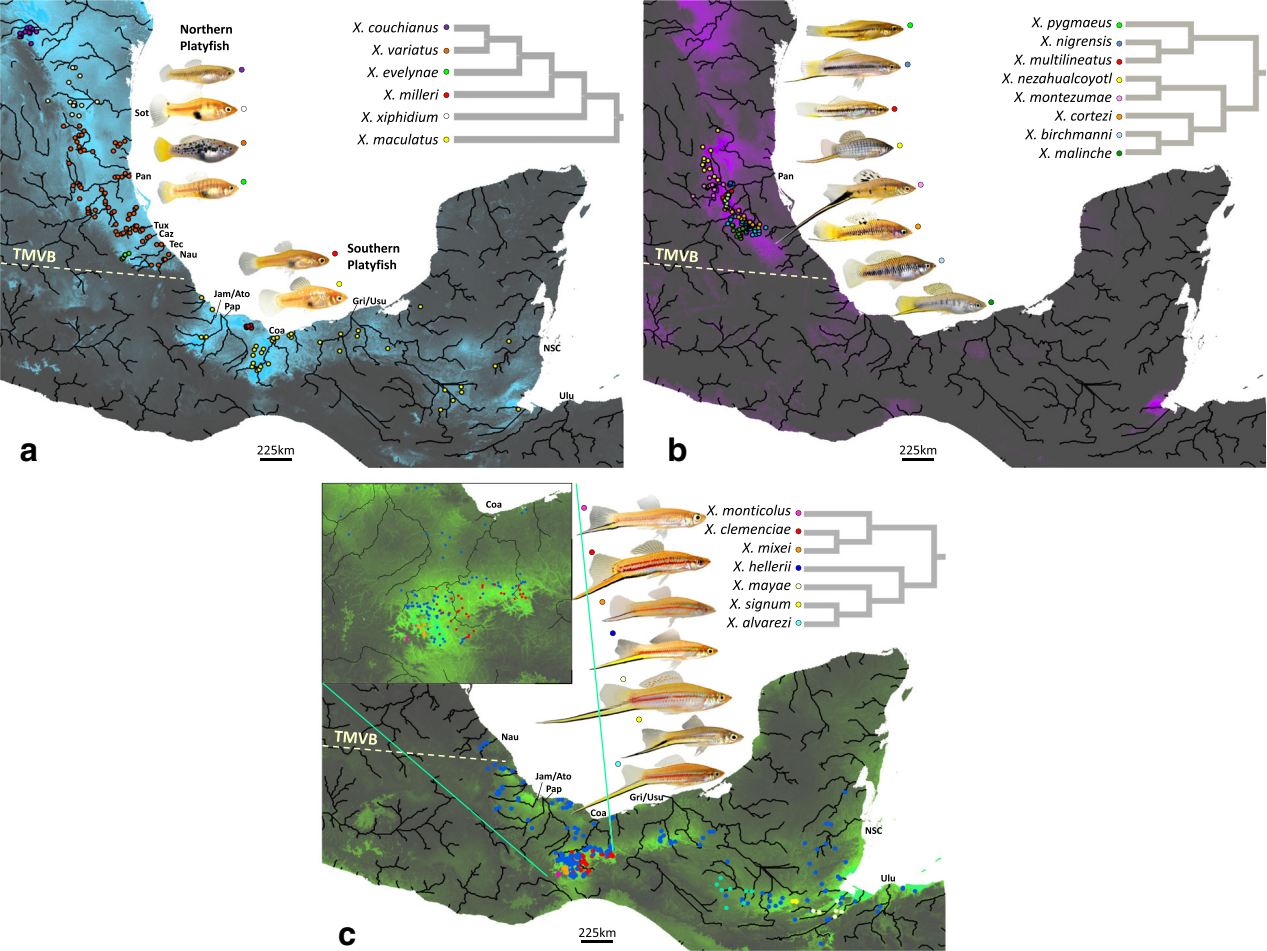
Distributional maps showing the occurrence points (colored dots) and cumulative environmental suitability for platyfishes (**a**), northern swordtails (**b**), and southern swordtails (**c**). Cumulative suitabilities were obtained by using the raster calculator tool in ArcMap 10.2.2 using the logistic outputs of the niche model for each species as produced according to procedures described in the methods. The resulting maps demonstrate where the environmental is not suitable for any species (black) to where the environmental is suitable for the greatest number of species of the clade (species-specific colors in panels **a-c**)

**Fig. 2:**
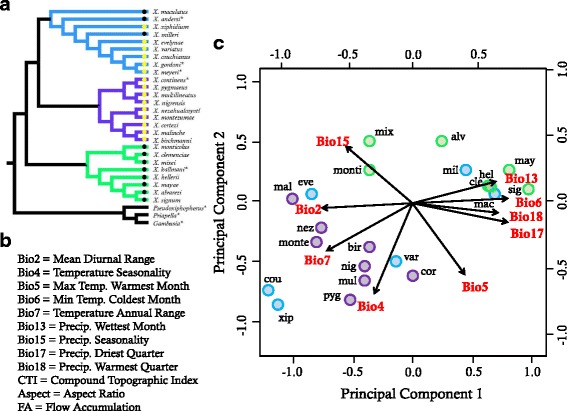
**a** Phylogenetic relationships in the genus *Xiphophorus*. Black (south) and yellow (north) dots on branches indicate the geographic location of species relative to the Trans-Mexican Volcanic Belt. Major clades are color-coded with platyfishes in blue, northern swordtails in purple, and southern swordtails in green. Species not included in niche modeling are indicated with asterisks. For a larger version of the phylogeny with estimated divergence times see Additional file 1. **b** List of the nine Worldclim and three hydrographic variables from Hydro1k used for niche modeling and phyloclimatic analyses. **c** Phylogenetic PCA for multivariate niche space. The x-axis plots the loadings for principal component 1 (PC1) and the y-axis for PC2. The arrows indicate the loadings of the individual environmental variables for each PC and arrows are labeled according to the Worldclim variable that they represent. Taxa are represented by the first three letters of the species name with the exception of *X. montezumae* (monte) and *X. monticolus* (monti). Hydrographic variables were excluded from the PCA (see methods)

Species of the genus *Xiphophorus* are well known as a model for investigating melanoma [33] and sexual selection (e.g., [34–36]). The evolutionary relationships among species have been the topic of considerable investigation and debate due to disagreement among phylogenies constructed from different data types (morphological *vs*. molecular: [26, 35, 37–39]), but the genus is generally comprised of three main, monophyletic clades: southern swordtails, northern swordtails, and platyfishes (Fig. 1a-c, Fig. 2a, Additional file 1). Discordance between mitochondrial and nuclear DNA phylogenies has prompted interest in hybrid speciation [40–43] and provided evidence of extensive historical gene flow among species [44]. The role of prezygotic reproductive isolation in speciation of *Xiphophorus* has been widely discussed and tested largely in the context of female preferences for and against sexually selected traits and chemical cues [27, 40, 45–50], but the extent of ecological divergence and its potential role in promoting speciation has not been investigated. Here, we used ecological niche modeling in a phylogenetic context to test for the roles of niche conservatism and divergence during diversification and to examine patterns of niche evolution across the major clades within *Xiphophorus*.

Specifically, we tested the prediction that if niche evolution (and potentially patterns of speciation) is associated with niche conservatism, then sister and closely related species within each of the major clades of *Xiphophorus* should have more similar niches than expected under a null model. Alternatively, if speciation is associated with niche divergence, then closely related species should have less similar niches than expected under a null model. We first used a hypothesis-testing approach, in which a null distribution of expected niche overlap values was generated by randomly sampling points within the distributions of two focal species, in order to compare to the observed niche overlap between those species as calculated from their niche models. Our results indicated contrasting patterns of niche conservatism and divergence in the swordtails and platyfishes, respectively. To further characterize patterns of niche evolution across the genus, we employed phylogenetically controlled analyses of multivariate (principal component analysis) and univariate niche space (ancestral tolerances and accumulation of disparity). These additional analyses allowed us to illustrate patterns of niche similarities and differences across the genus and to examine cases of divergence and convergence upon similar niche characteristics for single environmental variables. Based on the results of our hypothesis testing, we predicted that species would tend to cluster with other members of their clade in multivariate space and for tolerances of single environmental variables. However, following evidence of reduced niche similarity among platyfishes, we also predicted that platyfishes would exhibit greater variation along niche dimensions than either swordtail clade.

## Results

### Niche overlap & phylogeny

**Fig. 3:**
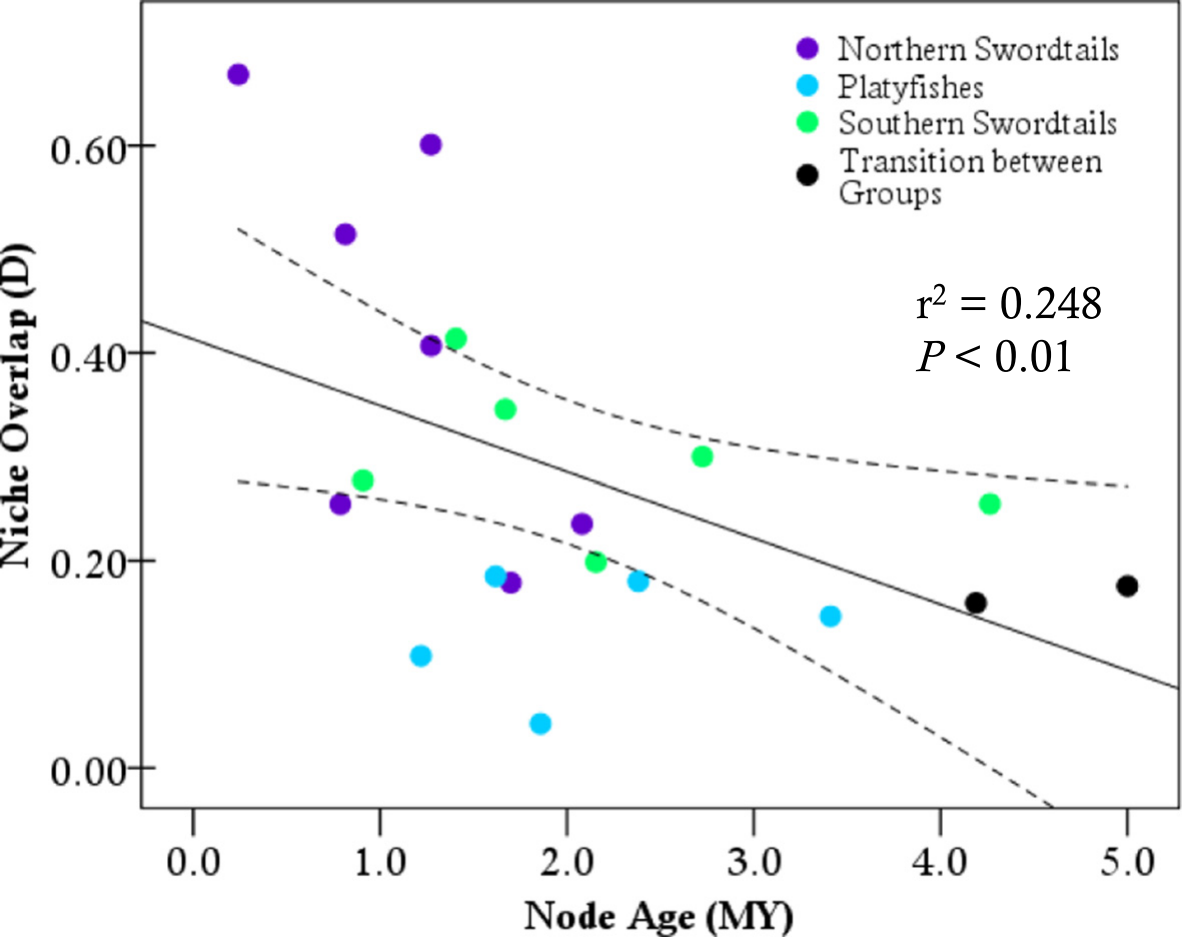
Results of the linear regression between node age and niche overlap. The significant negative slope indicates phylogenetic signal in niche overlap consistent with ecological divergence during speciation across the evolution of *Xiphophorus*. Dotted lines indicate 95 % confidence intervals

Schoener’s *D* and Warren’s *I* were used to broadly characterize patterns of niche similarity among species of *Xiphophorus* (Additional file 2). Values of *D* and *I* generally provided qualitatively similar results, although the *I* metric tended to give higher values than *D* for any given comparison (also see [19]). Within the three major clades, there was relatively low niche overlap among species (range, mean *D* ± SD; platyfishes: 0.004─0.535, 0.169 ± 0.168; northern swordtails: 0.100─0.660, 0.356 ± 0.133, southern swordtails: 0.064─0.560, 0.254 ± 0.134; Additional file 2). Niche overlap between species from different primary clades was even lower (mean ± SD; platyfishes *vs*. southern swordtails: 0.151 ± 0.133; northern swordtails *vs*. southern swordtails: 0.104 ± 0.067; Additional file 2), except for platyfishes and northern swordtails (0.246 ± 0.142), which had greater overlap than was observed among all platyfishes. There was a significant negative correlation between niche overlap at internal nodes and divergence time (Fig. 3), which is consistent with an accumulation of niche differences with increasing phylogenetic distance (Fig. 3). More importantly, eleven nodes exhibited niche overlap values outside of the 95 % confidence intervals for the regression. All nodes that exhibited significantly greater overlap than expected (*n* = 4) were within swordtail clades. A total of seven nodes exhibited less overlap than expected three of which were within swordtail clades, but the remaining four belonged to the platyfish clade (Fig. 3).

### Niche identity and background

Across the 64 pairwise species comparisons made within the three major clades of *Xiphophorus*, only two had equivalent niches (Additional file 2). Niche identity tests showed that *X. cortezi* had an equivalent niche to that of *X. pygmaeus* and *X. multilineatus*, though the latter two did not have equivalent niches. Background tests revealed that niche conservatism was intermediate between species in each of the two major swordtail clades, with 17/28 (61 %) and 9/21 (43 %) of comparisons exhibiting significantly greater niche overlap than the null expectation (in at least one direction) in the northern and southern swordtails, respectively. The more restrictive comparison of backgrounds only between sister species showed a strong pattern of conservatism, as all comparisons exhibited greater niche overlap than the null expectation. In contrast to the swordtails, the platyfishes tended to have much lower overlap than expected based on the available background environment. Only 2/15 (13 %) tests indicated some degree of conservatism. Most importantly, no sister species of platyfishes exhibited niche overlap greater than expected based on the available background conditions.

### Ancestral tolerance & phylogenetic PCA

While some climatic niche variables exhibited little variation across the genus (e.g., maximum temperature in the warmest month: 29.8–35.7 °C), there was considerable variation in others (Table 1). Temperature seasonality ranged from 13.7 to 41.4 °C, minimum temperature in the coldest month from 7.0 to 18.9 °C, and precipitation in the driest quarter from 5.8 to 20.5 cm. Interestingly, southern platyfishes had closer affinities to the niches of southern swordtails, and northern platyfishes more closely resembled the northern swordtail clade.

**Table 1:** Weighted means of the predicted niche occupancy for all environmental variables used in niche modeling and for all species of *Xiphophorus* included in our analyses. Worldclim variables related to temperature are reported in degrees Celsius and precipitation variables in centimeters. The Hydro1k variables retained for analyses were compound topographic index (CTI), aspect ratio (Aspect), and flow accumulation (FA). The species are grouped by their primary evolutionary clade and by their position in the phylogenetic tree (Fig. 2a). Species present in Fig. 2a but not in the table were not niche modeled due to too few occurrence points. Letters behind the platyfish species names indicate their position relative to the Trans-Mexican Volcanic Belt (TMVB) as north (N) or south (S). All northern swordtails are north and all southern swordtails south of the TMVB, respectively

		Worldclim variables									Hydro1k variables		
		Temperature (°C)					Precipitation (cm)						
Clade	Species	Bio2*	Bio4*	Bio5	Bio6	Bio7*	Bio13	Bio15*	Bio17	Bio18	CTI	Aspect	FA
Platyfishes	*X. maculatus*	9.9	19.9	33.8	17.7	16.1	37.8	6.5	16.0	48.6	763.6	23247.8	614.1
	*X. xiphidium*	13.7	40.5	34.0	8.4	25.6	14.4	6.9	5.9	25.0	596.0	21391.2	382.3
	*X. milleri*	9.6	19.8	32.5	16.3	16.2	38.8	7.1	13.7	43.4	589.2	19315.2	286.5
	*X. evelynae*	13.7	28.3	29.8	7.0	22.8	27.2	8.1	8.4	44.4	470.0	17405.7	265.1
	*X. variatus*	11.1	33.7	33.8	13.1	20.7	24.6	7.0	10.4	48.7	601.6	15223.5	852.3
	*X. couchianus*	13.2	41.4	32.5	7.2	25.3	13.7	7.0	5.4	23.1	541.9	13603.1	478.7
Northern Swordtails	*X. pygmaeus*	13.2	38.2	35.7	11.2	24.5	26.7	7.5	9.1	44.4	611.8	17190.5	563.4
	*X. nigrensis*	11.4	35.3	35.0	13.3	21.7	23.2	7.8	7.4	40.9	644.2	17252.0	315.2
	*X. multilineatus*	12.4	35.0	35.6	12.9	22.7	25.6	7.5	8.6	41.5	656.2	18054.4	607.0
	*X. nezahualcoyotl*	13.0	32.5	32.6	9.7	22.9	24.0	8.3	6.0	40.2	505.7	12885.8	251.4
	*X. montezumae*	13.2	33.5	32.9	9.6	23.3	23.8	8.5	6.1	42.0	525.1	14558.3	371.4
	*X. cortezi*	11.9	34.2	34.4	12.6	21.8	30.9	6.7	13.5	53.1	619.6	17123.5	754.2
	*X. birchmanni*	13.2	29.5	33.3	11.2	22.1	26.7	7.0	11.2	46.9	543.1	18249.3	267.4
	*X. malinche*	14.6	26.0	30.1	7.0	23.1	22.4	7.6	7.2	34.7	412.4	15254.1	80.8
Southern Swordtails	*X. monticolus*	11.9	19.7	32.9	14.3	18.6	25.9	8.4	6.9	29.0	654.2	19635.7	396.2
	*X. clemenciae*	10.5	19.1	33.8	17.0	16.8	42.9	7.1	17.4	47.3	601.9	17852.3	221.5
	*X. mixei*	11.3	17.2	32.5	15.2	17.3	25.8	8.8	5.8	25.3	564.6	16365.0	206.5
	*X. hellerii*	10.3	18.8	33.1	16.8	16.3	39.1	6.7	17.4	51.4	577.0	17241.4	68.4
	*X. mayae*	9.6	15.3	33.2	18.9	14.3	34.3	6.3	15.5	51.6	759.8	23619.7	1996.3
	*X. signum*	9.2	16.6	32.2	18.0	14.2	32.4	5.1	20.5	56.9	648.5	15546.0	153.6
	*X. alvarezi*	11.6	13.7	31.2	14.7	16.5	34.9	7.2	12.8	49.6	552.4	20222.3	485.8

*Variables are derived as follows:

Bio2 (mean diurnal range): mean of (monthly maximum temperature - monthly minimum temperature)

Bio4 (temperature seasonality): standard deviation of monthly mean temperature multiplied by 100

Bio7 (temperature annual range): maximum temperature in the warmest month - minimum temperature in the coldest month (i.e., Bio5 - Bio6)

Bio15 (precipitation seasonality): coefficient of variation of monthly mean precipitation

**Fig. 4:**
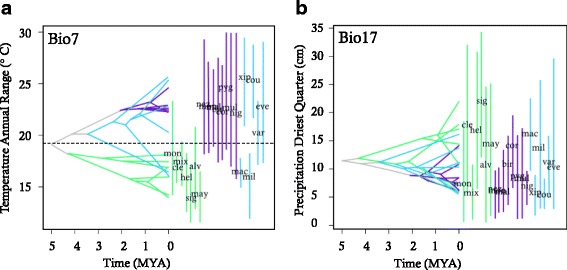
Ancestral tolerance plots (ATP) for annual temperature range (**a**) and precipitation of the driest quarter (**b**). Taxa are indicated by the first three letters of the species name and colored by primary clade corresponding to Fig. 2a. The ATPs for annual temperature range illustrate the conserved nature of tolerances within the swordtail clades and deep divergence within the platyfishes corresponding to the biogeographic division by the Trans-Mexican Volcanic Belt (TMVB). The dotted line in panel **a** denotes the separation north (above) and south (below) of the TMVB. The plot for precipitation in the driest quarter demonstrates a case where ATPs exhibited considerable overlap among the major clades and where some sub-clades diverged from their closest relatives (e.g., *X. monticolus* and *X. mixei* from the rest of the southern swordtails). Note that no such separation between species on either side of the TMVB can be drawn for panel **b**. Plots of ATP for all twelve variables can be found in Additional file 3

In ancestral tolerance plots (ATPs), branches for any two species can be traced back to a shared ancestral niche, and crossing of branches is indicative of divergent evolution in parameter space. For most of the environmental variables used in our niche models, the ATPs of *Xiphophorus* indicated relatively conserved niche evolution within each of the three main clades (e.g., Fig. 4a; Additional file 3). Despite the general pattern of niche similarity, there are a variety of instances of species diverging from members of their own clade (Fig. 4b) and converging to a tolerance more similar to that of members of a more distantly related clade (Additional file 3). One consistent pattern seen in several ATPs is the divergence of *X. maculatus* and *X. milleri* from the rest of the platyfishes, with a concomitant convergence on the niche space of the southern swordtails (bio2, bio4, bio6, bio7, and bio13; Additional file 3), which is consistent with divergence of platyfishes south and north of the TMVB. For species occurring south of the TMVB, all swordtail and platyfish niches were characterized by low temperature seasonality, a smaller annual temperature range, and warmer minimum temperatures. Species occurring north of the TMVB had niches with greater temperature seasonality and annual range and lower minimum temperatures. In contrast with the other three platyfishes north of the TMVB, *X. variatus* exhibited an ancestral tolerance for warmer minimum temperatures. Ancestral climate tolerances related to precipitation were more variable and exhibited more overlap among the major clades compared to temperature. For all four precipitation variables, there were numerous instances of branch crossings, suggesting convergence upon similar precipitation parameters across the three main clades. The most northern platyfishes in our analyses, *X. couchianus* and *X. xiphidium*, appear to have diverged into the driest regions of any *Xiphophorus*. Another interesting pattern is observed within the *X. clemenciae* clade of the southern swordtails. Despite the fact that all three members of this clade inhabit a similar geographic region (the upper reaches of the Coatzacoalcos drainage), *X. mixei* and *X. monticolus* have largely diverged from the rest of the southern swordtails and even from their sister species, *X. clemenciae* (bio13, bio17, bio18; Additional file 3).

As with the ATPs, the phylogenetic PCA indicated a general separation between species north and south of the TMVB, where the distributions of species south of the TMVB were influenced most by precipitation and minimum temperature in the coldest month, and those of species north of the TMVB were influenced by the remaining temperature variables (Fig. 2b-c). The phylogenetic MANOVA confirmed significant differences in the niches of species on either side of the TMVB (*F*_4,16_: 38.4, *P* < 0.001). An additional pattern supported by the pPCA was the difference of the platyfishes *X. maculatus* and *X. milleri* from the rest of the platyfish clade, indicating a strong divergence in platyfish niches. Variation in maximum temperature and precipitation seasonality was related primarily to niche differences within clades, and high precipitation seasonality was particularly characteristic for the distribution of the sister species, *X. mixei* and *X. monticolus*.

### Niche disparity

**Fig. 5:**
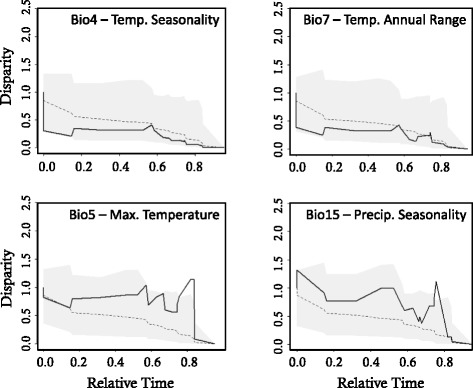
Observed disparity (solid line) was significantly lower than levels expected under a null model (dotted line) of unconstrained evolution for Bio4 and Bio7. The lack of disparity was associated with a significant (*P* < 0.05) signal of conservatism within the major clades corresponding to divergence ~5MYA between the southern swordtails and the rest of *Xiphophorus*. Most other variables, for example Bio5 and Bio15, exhibited zero or positive disparity through time with a variety of nodes exhibiting significant divergence within sub-clades corresponding to peaks in disparity in the plots. Gray shading denotes 95 % confidence limits

The relative disparity plots and associated MDI values reveal variation in patterns of niche evolution among environmental variables (Additional file 4). Temperature variables generally revealed a pattern of early conservatism within the major clades (divergence among clades), followed by increases in disparity suggesting subsequent evolution within subclades. In particular, temperature seasonality (bio4) and temperature annual range (bio7) exhibited MDI values below the expectation under Brownian motion (Fig. 5). Consistent with early divergence among clades, the node delineating the split between the southern swordtails and the rest of *Xiphophorus* exhibited significant divergence with respect to temperature seasonality compared to expectations under a Brownian model (*P* < 0.05). In contrast with other temperature variables, maximum temperature in the warmest month (bio5) did not exhibit the same pattern of early divergent evolution among clades, but rather showed positive disparity through time including a sharp increase in disparity around 1 MYA (0.8 relative time; Fig. 5). This pattern is indicative of evolution within subclades, and in fact, multiple nodes exhibited significant divergence for maximum temperature (*P* < 0.05). The first divergence in maximum temperature was associated with the split between the lowland and highland congeners *X. signum* and *X. alvarezi* in the southern swordtails. A second significant divergence event occurred in the northern swordtails between *X. pygmaeus* and its two closest congeners (*X. multilineatus* and *X. nigrensis*). Several other marginally non-significant peaks can be seen in the disparity plot for maximum temperature (Fig. 5), including two simultaneous events in the northern and southern swordtails at ~1.7 MYA (0.66 relative time): the divergence of the widely distributed *X. hellerii* (*P* = 0.054) and its sister clade, as well as the divergence of the clade uniting *X. birchmanni*, *X. cortezi*, and *X. malinche* (*P* = 0.074). Lastly, the peak at ~1.25 MYA (0.75 relative time) was associated with divergence of the northernmost platyfish, *X. couchianus*, from the rest of the platyfishes (*P* = 0.062).

Disparity plots for precipitation variables generally showed a pattern of positive MDI indicating greater evolution within subclades (e.g., bio15; Fig. 5). Precipitation seasonality (bio15) and precipitation in the warmest quarter (bio18) had the strongest signature of evolution within subclades (Additional file 4). Specifically, four nodes ranging from 0.67-0.74 relative time (~1.5 MYA), and spread across all three major clades of *Xiphophorus*, exhibited significant divergence for precipitation in the warmest quarter (*P* < 0.05). All precipitation variables showed large increases in disparity around 2.5 MYA, with three of four precipitation variables reaching their highest levels of disparity at that time point.

## Discussion

Evolutionary diversification of swordtails and platyfishes has been markedly shaped by sexual selection [27, 40, 50] and widespread introgressive hybridization [44]; yet, the importance and extent of ecological divergence in the genus *Xiphophorus* has remained largely unstudied. Our analysis of ecological niches across this group indicated contrasting patterns of niche evolution among the three major lineages. Hypothesis testing revealed a strong tendency of niche conservatism as indicated by significant similarity in niches compared to background environments among closely related species of the northern and southern swordtail clades. In contrast, species in the platyfish clade have undergone a high degree of niche divergence. These general patterns were well supported in all analyses and were discernible in tests of multivariate niche space (e.g., phylogenetic PCA) as well as in single environmental variables (e.g., ancestral tolerances). Furthermore, patterns of niche variation in *Xiphophorus* were strongly bisected by the Trans-Mexican Volcanic Belt, illustrating an important association between biogeography and patterns of niche evolution at broader taxonomic scales. This pattern was particularly evident in platyfishes on either side of the TMVB, which showed greater niche similarities to and shared ancestral tolerances with swordtails exhibiting a similar geographic distribution, rather than with phylogenetically related platyfishes. The consistency of the results from multiple analyses of niche evolution in *Xiphophorus* indicate that both divergence and constraint have accompanied diversification, which highlights avenues for future research on the roles of ecological adaptation and constraint in speciation. Our results also support previous studies in insects, amphibians, birds, and mammals in underscoring the importance of taxonomic scale for making inferences about divergence and conservatism in niche evolution [19, 51]. For example, a focus on either swordtail group alone would have suggested high niche conservatism during the diversification of *Xiphophorus* fishes, while a focus on platyfishes would have provided strong evidence towards niche divergence. In reality, the relative importance of niche conservatism and divergence during diversification can vary even among closely related species.

### Has niche divergence accompanied speciation?

The extent and importance of ecological divergence during speciation remains an open question in ecology and evolution [52]. Studies of climatic niche evolution have reported evidence of both divergence [20, 53] and conservatism [19, 51, 54, 55]. We uncovered a significant correlation between phylogenetic distance and niche overlap. This pattern is consistent with the accumulation of ecological differences over evolutionary time. However, this does not in itself allow for inferences to be made about the roles of niche conservatism and divergence during diversification. Examining the outliers from this correlation indicated that nodes exhibiting greater niche overlap than expected based on their age (i.e. potential conservatism) only occurred within swordtail clades. The nodes exhibiting less overlap than expected (i.e., potential divergence) mostly belonged to the platyfish clade (4 of 5 platyfish nodes had reduced overlap). Potential niche conservatism in swordtails and niche divergence in platyfishes was also supported by other analyses. Background similarity tests indicated that closely related species had higher levels of niche overlap than expected by chance, with 63 % of swordtail sister species exhibiting significant niche conservatism. Conservatism remained high at broader taxonomic scales within these two clades as 61 % (northern swordtails) and 43 % (southern swordtails) of pairwise comparisons indicated significantly greater overlap compared to a null model, and only 2 (7 %) and 3 (14 %) tests, respectively, indicated significantly divergent niches. However, niche identity tests revealed that only two pairwise comparisons within northern swordtails had equivalent niches. Low niche equivalency indicates that while swordtails are characterized by niche conservatism (niches are significantly more similar than their environmental backgrounds), speciation has been accompanied by shifts in climatic niches. In contrast to the swordtails, platyfishes exhibited no equivalent niches and almost no conservatism. Among comparisons restricted to sister species of platyfishes, none of the tests indicated niche conservatism. Across all pairwise comparisons of platyfishes, only 2/15 (13 %) comparisons had niche overlap values greater than expected, but 9/15 (60 %) exhibited significant niche divergence with less niche overlap compared to the null model. Our results for the two swordtail clades tend to coincide with previous work showing that 80-90 % of sister species exhibit niche conservatism in analyses of Mexican butterflies, birds, and mammals [19]. In that context, it becomes evident that diversification in platyfishes is characterized by much greater niche diversification, perhaps suggesting that adaptation to divergent environmental conditions is an important driver of platyfish speciation. Our results are similar to findings on other North American fishes, which generally suggest that some clades are characterized by niche conservatism while others are not [56]. Similarly, asymmetries in the degree of niche divergence and conservatism have also been observed in other non-fish systems, albeit at broader taxonomic scales than those in this study. For example, estimates of niche conservatism based on geographic overlap of terrestrial vertebrates and conservatism in habitat affinities in marine invertebrates have each exhibited variation among very divergent taxa [57, 58]. Our data show that such asymmetries among taxa can be apparent at much smaller geographic and taxonomic scales.

The cause for the contrasting evidence for niche divergence and conservatism in different clades of *Xiphophorus* fishes remains unclear. Given that the platyfishes broadly overlap the geographic distributions of swordtails and that both swordtail clades are characterized by high conservatism suggests that intrinsic rather extrinsic factors may play a role in niche evolution for these respective groups. In general, there may be three non-mutually exclusive hypotheses that could explain the observed pattern: (1) The documented differences among clades may be an artifact of incomplete taxon sampling. The only taxa not included in our analyses are those that are highly endemic with too few, independent occurrence points to conduct niche modeling. It is unlikely that we have underestimated divergence in the two swordtail clades, because our analyses were only missing one species each from the northern (*X. continens*) and the southern (*X. kallmani*) swordtail clades. *Xiphophorus continens* is closely related to *X. pygmaeus* [39, 44], and like all other northern swordtails, it occurs in the Rio Panuco drainage [59]. *Xiphophorus kallmani* is endemic to the Laguna de Catemaco, which is within the geographic distribution of *X. hellerii* [60]. Our analyses were missing three species of platyfishes. *Xiphophorus gordoni* and *X. meyeri* are closely related to *X. couchianus* comprising a single, monophyletic clade. All three species occur in tributaries of the Rio Grande basin and have restricted ranges in an arid region of northern Mexico that is geographically removed from the remainder of the genus [60]. Accordingly, the three species may inhabit similar environments and exhibit similar niches to one another, but as a group are likely divergent from other platyfishes. Finally, *X. andersi* is a more basal platyfish [39, 44] that occurs south of the TMVB, but inhabits a high-elevation, headwater habitat that is distinct from its closest relatives *X. maculatus* and *X. xiphidium* [60]. (2) Differences in the degree of niche conservatism between the swordtails and platyfishes could be a consequence of the environmental variables included in this study. Niche modeling focused on parameters that characterize climatic niches based on distributional patterns. Even in the absence of niche divergence in climatic niche space among northern and southern swordtail species, ecological divergence may have occurred along other biotic or abiotic niche dimensions [61]. This is a particularly intriguing problem for the northern swordtails. All northern swordtail species occur in the Rio Panuco drainage, and some species occur sympatrically within the same habitats [60]. This provides an opportunity for future studies to test for potential ecological differentiation among species along other niche axes. (3) The relative importance of niche divergence and conservatism during diversification may be related to the strength of sexual selection. Swordtails commonly have exaggerated male traits, including elaborate caudal extensions [46] and enlarged dorsal fins [25]. In addition, there is a wealth of evidence for the presence of female mate choice for a variety of male traits in swordtails [25, 34–36, 62, 63], but see [64]. Nonetheless, it is difficult to say whether platyfishes are characterized by weaker sexual selection. Even though platyfishes generally do not have the sword ornament, females exhibit preferences for swords [65], enlarged dorsal fins [64, 66], and platyfishes tend to have a much greater variety of genetic pattern and color traits compared to swordtails [67, 68]. Whether differences in niche divergence and conservatism within *Xiphophorus* is due to a constraining effect of sexual selection on niche evolution in swordtails remains to be investigated, but other studies have found evidence of natural and sexual selection reinforcing or constraining one another during diversification [69–71].

### Biogeography and climatic niches

A consistent trend observed in multiple analyses was the broad separation in the niches of species on either side of the TMVB. For example, phyloclim analyses revealed that ancestral tolerances of platyfishes overlapped those of both swordtail clades. Specifically, the two southern platyfish, *X. maculatus* and *X. milleri*, tended to have tolerances similar to the southern swordtails (for example, Fig. 4a). Geographic isolation during speciation likely facilitates the accumulation of ecological differences. Biogeography and associated vicariance is therefore important for understanding evolutionary patterns of species niches [72, 73]. Studies in plants have suggested that biogeographic breaks in species distributions promote diversification and increases in trait disparity [20, 74], and our results generally agree with such patterns. The TMVB is a major biogeographic break for freshwater fishes [75, 76] and was associated with strong divergence between the niches of the ancestral southern swordtails and the northern swordtails as well as divergence within the platyfishes. In addition to ancestral tolerances, this pattern was also recovered in the phylogenetic PCA and MANOVA analyzing variation in multivariate niche space (Fig. 2c).

Previous work on the role of biogeography in niche evolution in Mexican butterflies, birds, and mammals found considerable niche conservatism between sister species on either side of the Isthmus of Tehuantepec, an arid region separating more moist, montane regions [19, 77]. Though a direct comparison to those studies is difficult here, particularly because sister species of *Xiphophorus* generally do not inhabit both sides of the TMVB, our data revealed significant differences in the niches of species living on opposite sides of the TMVB even after controlling for phylogenetic distance. If niche conservatism were a strong factor for niche evolution of sister species, we would have expected *X. maculatus* to have stronger niche similarity with *X. xiphidium*, and *X. couchianus* to have similarity with *X. variatus*, their closest phylogenetic relatives. Instead, *X. maculatus* and *X. couchianus* exhibited significant similarity only with their closest geographic congeners. Consequently, major biogeographic barriers like TMVB may be critical in shaping the evolution of ecological niches.

### Lineages in unique environments

Despite high degrees of niche conservatism in swordtails, niche identity comparisons revealed that closely related species almost never have equivalent niches. Analyses of the accumulation of disparity and ancestral tolerances provided insight into specific instances of when and for what variables closely related species have diverged, and several examples of divergence and convergence were also apparent in multivariate space (phylogenetic PCA): (1) *Xiphophorus monticolus* and *X. mixei* have diverged from their sister species, *X. clemenciae,* for all precipitation variables despite close geographic proximity. (2) The northernmost platyfishes, *X. couchianus* and *X. xiphidium*, exhibited divergent tolerances for several temperature and precipitation variables compared to the rest of the platyfishes and were clustered in a unique area of multivariate space. (3) The niches of the northern swordtail clade containing *X. pygmaeus* and its closest relatives have diverged towards higher maximum temperatures (bio5; Additional file 3). (4) Most interestingly, members of each of the main clades (*X. alvarezi* in the southern swordtails, *X. malinche* in the northern swordtails, and *X. evelynae* in the platyfishes) have converged on similar high-elevation niches characterized by cooler maximum temperatures (bio5; Additional file 3). The convergence between *X. evelynae* and *X. malinche* was particularly apparent in the pPCA, and the strong divide in niche space between species north and south of the TMVB is likely the reason that *X. alvarezi* did not cluster with these two species, even though it was clearly divergent from its closest relatives (*X. mayae* and *X. signum*).

Species that occupy distinct portions of niche space will provide unique models to understand the relative roles of adaptation and plasticity that underlie differences in observed niches based on distributional data. This remains a major task for the field, because, adaptive divergence cannot be inferred from correlative modeling approaches alone. One well-documented case in *Xiphophorus* suggests that species are adapted to local climatic conditions. Replicated hybrid zones between *X. birchmanni* and *X. malinche* occur along an elevational and thermal gradient in multiple streams of the Rio Panuco drainage [78]. Physiological tolerances to thermal extremes in wild-caught and common-garden fish together with gene expression analyses demonstrated the role of physiological adaptation to local thermal environments as a key factor in the maintenance of the geographic structure of hybrid zones [79].

It remains unclear whether adaptation to novel niche space promotes vicariance or whether vicariance followed by adaptation accelerates or facilitates the maintenance of reproductive isolation. A study on North American Monkeyflowers suggests that speciation is often initiated by small populations occupying novel niche dimensions [80], typically through colonization of the new environment followed by reproductive isolation (budding speciation). Budding speciation could therefore be one mechanism by which ecological divergence occurs. The uplift of mountain ranges, which slowly isolates upstream populations from downstream congeners, may be a special case of budding speciation. Such isolation by orogeny has been implicated for some species of *Xiphophorus* [60] and may explain replicated instances of divergence in novel niche dimensions.

## Conclusions

The relationship between niche evolution and speciation remains widely studied, because conservatism and divergence might equally promote the emergence of new species [51]. In our study, the degree of niche conservatism observed across the genus *Xiphophorus* varied markedly among clades. An equally interesting and important question stems from this pattern: what gives rise to such asymmetry in niche evolution within a single genus? Additional studies are needed to better understand the relative frequency of niche divergence and conservatism during speciation and the extent to which heterogeneous patterns of niche evolution occur in other taxa as we observed in *Xiphophorus*. Future studies will need to combine analyses of species distributions and ecological niches with functional assays of relevant physiological and morphological traits that ultimately shape organismal fitness. This is a substantial task considering that a persistent problem for fishes and other aquatic organisms is to model the functional ecological and physiological niche based on landscape level environmental data that has more predictable effects on organismal performance. Whereas temperature variation can give rise to explicit testable hypotheses related to temperature adaptation, the effects of many variables that are currently available and commonly used on ecological niche modeling for aquatic organisms, including various precipitation metrics, are often difficult to interpret, and most parts of the world do not have the necessary infrastructure nor historical data records to consider the use of more relevant environmental variables, such as dissolved oxygen concentrations, in models that span large spatial and taxonomic scales.

## Methods

### Distributional patterns and occurrence records

As a group, the distribution of the genus *Xiphophorus* stretches more than 2000 km from northeastern Mexico to Guatemala and Honduras [60], including broad elevational gradients and a wide range of habitats from small springs and headwater streams to large rivers and a water-filled caldera forming the Laguna de Catemaco in the Mexican state of Veracruz [60]. Numerous members of the genus inhabit apparently similar stream habitats with varying degrees of geographic isolation, and the three main clades of *Xiphophorus* exhibit both differences and commonalities in their distributional patterns (Fig. 1a-c).

The most basal divergence in *Xiphophorus* gave rise to the southern swordtails and the common ancestor of platyfishes and northern swordtails [43]. The southern swordtails have the most southern range and occur from southern Mexico to northern Guatemala and Honduras. There is considerable variation in the geographic ranges of individual species. Within southern swordtails, species of the *X. clemenciae* clade (*X. clemenciae*, *X. mixei*, and *X. monticolus*) are only found in upland reaches of the Rio Coatzacoalcos in Mexico. Four additional species (*X. alvarezi*, *X. mayae*, *X. signum,* and *X. kallmani*) are scattered across several drainages from southern Mexico through northern Honduras, but like members of the *X. clemenciae* clade, each has a relatively restricted range. In contrast to all other southern swordtails, *X. hellerii* is widely distributed from the Trans-Mexican Volcanic Belt (TMVB) to Honduras. The northern swordtails are found to the north of the TMVB and are almost entirely restricted to the Rio Panuco drainage. Each species has a relatively restricted distribution in different sub-drainages, or they segregate along elevational gradients within sub-drainages [60]. The TMVB therefore broadly defines the geographic separation between the northern and southern swordtails. Interestingly, the platyfishes span both sides of the TMVB. The platyfish, *X. maculatus*, is basal to other species of platyfishes and is widely distributed to the south of the TMVB, suggesting that southern Mexico was a likely origin for the platyfishes. Two additional platyfishes inhabit the region south of the TMVB; *X. milleri* occurs in the Laguna de Catemaco and its tributaries (sympatric with *X. kallmani* also endemic to Catemaco), and *X. andersi* is known from a single locality in the state of Veracruz. More recently derived platyfishes (*X. couchianus*, *X. gordoni*, and *X. meyeri*) have colonized spring habitats of the Rio Grande drainage in the northern Mexican states of Coahuila and Nuevo Leon, which are geographically isolated from other species of *Xiphophorus*. The remaining platyfishes north of the TMVB vary in their distributions, ranging from highly restricted headwaters of a single basin (*X. evelynae*) to numerous streams of a single drainage (*X. xiphidium*), and being widely distributed from the Rio Nautla to the Rio Soto La Marina (*X. variatus*). Herein, we follow the vernacular of Kallman and Kazianis [60] in referring to northern and southern platyfishes based on their geographic distributions relative to the TMVB.

We obtained species occurrence data for all *Xiphophorus* species from the Fishnet2 Portal (http://fishnet2.net), the literature [59, 60, 81–86], and field collections in Mexico and Honduras conducted by the authors. All occurrence records were vetted against the literature to ensure that occurrences for each species fell within their documented, native distributions [59, 60], and non-native records were removed from the dataset. For each species, we also removed occurrence records that were <1 km apart to match the spatial resolution of environmental variables (see below).

### Time-calibrated phylogeny

In order to obtain a time-calibrated topology for phlyoclimatic analyses, we used the maximum clade credibility topology of Jones et al. [43], which was derived from genome-wide restriction-site associated DNA sequencing. This phylogeny includes all 26 described swordtail and platyfish species. Prior to time calibration, taxa with too few occurrence records (<5) for ecological niche modeling were manually pruned using Mesquite v2.91 [87]. Divergence times were estimated using the ‘chronos’ command in the package *ape* [88] in R. In this method divergence times are estimated with a penalized likelihood approach, and a correlated substitution rate was employed [89]. Generating a time-calibrated phylogeny for *Xiphophorus* is difficult due to a lack of fossil calibration points. Discordance between the mitochondrial and nuclear phylogenies in *Xiphophorus*, likely due to historical gene flow and introgressive hybridization [44], also precludes use of a mitochondrial phylogeny and molecular clock dating. Although the relative divergence times within the genus *Xiphophorus* is most important for the analyses and interpretations herein, the estimated absolute divergence times are in general agreement with several lines of evidence.

The timing of geological events in Mexico is relatively well known, including the closure of the TMVB, a key geographic barrier to dispersal. The final uplift of the TMVB towards Punta El Morro on the Gulf Coast of Mexico occurred approximately 5 million years ago (MYA). Prior studies of Mexican fishes have used the timing of this geological event to calibrate divergence times in cichlids and other poeciliids [75, 76, 90], due to the fact that it represents a major biogeographic break in the distributions of many fish taxa, including *Xiphophorus* [60]. We therefore constrained the age of the initial divergence in *Xiphophorus* to a minimum of 4.5 MYA and a maximum of 5 MYA, assuming correlated substitution rates among clades. This approach placed the divergence of *X. milleri*, endemic to the Laguna de Catemaco, between 1.5–2 MYA (Additional file 1), which is consistent with estimated geological dates for the lake [91]. The estimated age of *X. milleri* was also consistent with the estimated age of another livebearer endemic to the lake, *Poeciliopsis catemaco,* as calculated using a molecular clock approach with mitochondrial DNA [92].

### Ecological niche modeling

We assembled a set of coverages for 24 environmental variables from the Worldclim (http://www.worldclim.org) and Hydro1k (https://lta.cr.usgs.gov/HYDRO1K) project databases. Data layers were downloaded at 30 arc-seconds (~1 km^2^) resolution. The Worldclim dataset is composed of nineteen bioclimatic variables related to temperature and precipitation, and the Hydro1k dataset is composed of five variables related to hydrography [93, 94]. Variables in these datasets have been successfully used in previous studies analyzing distributions of freshwater fish species with ecological niche modeling, including several poeciliids [79, 95–97]. All data layers were clipped to the area of study encompassing the native distribution of the species included in this study (see Fig. 1a-c).

Ecological niche models were constructed for each species using MAXENT v3.3.3 [98]. A few narrowly endemic species in our analyses had limited occurrence data (5–8 points) due to small ranges, but MAXENT has been shown to perform better than other methods for constructing ENMs when the number of occurrence points is low (5–10: [99]). Nonetheless, some narrowly endemic species of *Xiphophorus* have such restricted ranges (e.g., limited to a single spring) that there were not enough occurrence points for modeling procedures (<5: *X. andersi*, *X. continens*, *X. gordoni*, *X. kallmani*, *X. meyeri*). These species were thus excluded from niche analyses. Although MAXENT performs well when variables are highly correlated, reducing variables to those that are ecologically relevant and non-redundant facilitates interpretation of the resulting models [100] and minimizes the potential for model over-fitting [101]. In order to remove redundant variables, we conducted a principal components analysis using the Spatial Analyst tools available in ArcMap v10.2.2 to construct a correlation matrix for the nineteen bioclimatic and five hydrographic variables. For variables with a correlation of *r* > 0.9, we retained only a single variable, and preferentially retained variables that measure extremes over those that measure averages [17, 79]. Environmental extremes are more likely to affect the range limits of species, as they provide greater opportunity for selection on physiological tolerances [4]. This procedure left us with twelve environmental variables for analyses (9 Worldclim and 3 Hydro1k; Fig. 1b,c and Table 1). We used auto features and recommended default settings in MAXENT and increased the maximum number of iterations to 1000. To gauge the sensitivity of each species ENM to the samples used to train the model and to test the predictive ability of the models, we performed 10 replicate runs for each species model using a different random seed and subsampling with 59 % of samples allotted for training and 41 % for testing. For each species, we examined the mean Area Under the Receiver Operating Curve (AUC) across the 10 replicates and considered a mean AUC value ≥ 0.7 as evidence that the model had sufficient discriminatory ability [102]. For all species, the mean AUC was ≥ 0.89. The final ENM for each species was constructed using all occurrence points to train the model and the Minimum Training Presence threshold to determine suitability across the geographic extent of the study region [103].

### Niche evolution analyses

Niche models were used to calculate niche overlap among all pairwise species based on Schoener’s *D* and Warren’s *I* [19] in R using the package *phyloclim* (http://www.christophheibl.de/Rpackages), which was used for all subsequent analyses unless otherwise stated. Both *D* and *I* range from 0 (no overlap) to 1 (complete overlap). Because low niche overlap may be a correlated with phylogenetic distance as a consequence of neutral evolution rather than niche differentiation through divergent selection, we tested for phylogenetic signal in patterns of niche overlap using the age.range.correlation (ARC) command and used SPSS v17.0 (IBM Corp.) to generate 95 % confidence intervals around the correlation. This procedure applies a linear regression of node age to niche overlap to test whether there is a relationship between time since divergence and niche overlap at all nodes of the phylogeny. No correlation would indicate a random association between node age and niche overlap, whereas either a significant negative or positive correlation indicate phylogenetic signal in niche evolution. While the correlation itself does not indicate niche conservatism or divergence, examining outliers can help identify nodes that exhibit greater or lesser niche overlap than expected for their age.

In order to explicitly evaluate the influence of niche conservatism and divergence during *Xiphophorus* diversification, we tested hypotheses about niche overlap using the niche identity and background tests [19]. These tests quantify niche similarity between species in the context of a null model, enabling inferences of niche conservatism and divergence. Because tests for niche conservatism are most appropriate for investigating potential shifts in ecological niche dimensions among closely related species, we restricted our tests to pairwise comparisons within each of the three primary clades. Niche identity tests evaluate whether the niches of a given pair of species are identical. However, a rejection of niche identity between two species may be driven by differences in available environmental conditions within the distributions of the two species. Background similarity tests provide an additional comparison by testing whether observed niche overlap between two species is greater than the overlap expected based on the environmental conditions available to each species (i.e., niche conservatism). Alternatively, observed overlap may be less than that expected base on the environmental background of the species (i.e., niche divergence). Background similarity analyses generate random “pseudolocality” points within the observed distributions of two species and then test whether the observed niche overlap is more or less than would be expected given the available environmental backgrounds of the two species. As in previous studies [104–106], we used 100 replicates and a confidence interval of 0.95 to assess significance.

In order to trace the evolutionary history of species niches through time, we produced predicted niche occupancy (PNO) profiles [20]. For a given species, this approach uses probability distributions output by MAXENT to quantify the occupancy of that species across all niche space (with the probabilities summing to 1). This process was repeated to generate PNOs for each species and all environmental variables. The PNO profiles were then used to reconstruct the ancestral tolerances for each environmental variable. These ancestral tolerances can be plotted similar to a phylogenetic tree and allow inference of divergent and convergent evolution along a particular niche axis. The ancestral tolerances were constructed using a maximum likelihood approach under a model of Brownian motion evolution and using 100 random samples from the PNO profiles of all taxa as implemented in *phyloclim*. In contrast to prior methods that reconstructed ancestral tolerances with respect to only extreme (maximum or minimum) or mean values of environmental variables, this approach reconstructs the entire distribution of ancestral environmental tolerances and can better account for intraspecific niche variation during ancestral state reconstruction [20]. Finally, we calculated the weighted means of the PNOs for each species and environmental variable. Because ancestral tolerance analyses produce a separate plot for every environmental variable, we used a phylogenetic principal components analysis (pPCA: [107]) on the PNO weighted means to summarize occupancy of multivariate niche space across the genus using the package *phytools* [108]. The weighted means of predicted niche occupancy (Table 1), ancestral tolerance plots (Fig. 4 and Additional file 3), and a preliminary PCA all indicated that species niches exhibited relatively little variation in hydrographic variables, rendering them uninformative for explaining differences among species. These variables were therefore excluded from the final PCA. To examine patterns of niche evolution associated with the TMVB biogeographic divide, we conducted a phylogenetic multivariate analysis of variance (MANOVA) using the first four phylogenetic principal components obtained from the phlogenetic PCA. The analysis was run in R with the package *geiger* [109] by comparing observed effects of position relative to the TMVB (north or south) to a null distribution of the *F*-statistic generated from 1000 simulations under a Brownian motion model.

As a final approach to examine niche evolution, we constructed relative disparity plots [110]. This approach quantifies the extent to which disparity in the niche is distributed within or among subclades and illustrates temporal patterns in the accumulation of niche disparity among clades. The x-axis of a disparity plot indicates relative evolutionary time ranging from 0 (root of the phylogeny) to 1 (present day). The y-axis describes the observed disparity and the disparity expected under an unconstrained, Brownian model of evolution. Disparity was calculated as the average squared pairwise distance between taxa with respect to the mean predicted niche occupancy for each interior node and standardized by the disparity observed across the entire clade. We then calculated a disparity index based on environmental variables following the definition of the Morphological Disparity Index (MDI: [110, 111]), which allows comparison of the observed disparity values to values expected under a Brownian motion model of evolution, using 1000 simulations and a confidence interval of 0.95. Negative MDI values ─ where disparity is less than the expected value under the Brownian model on the disparity plots ─ indicate that disparity is distributed among subclades, consistent with conservatism within more inclusive clades and divergence among more inclusive clades. Positive MDI values indicate that disparity is within subclades, consistent with divergence among subclades. In order to test whether any nodes exhibited MDI that deviated significantly from its simulated null distribution, we calculated the rank of the observed disparity value for the node within the simulated values as described in Swenson [112]. Disparity and MDI analyses were conducted in R using the package *geiger* [109].

## Additional files

Additional file 1:
**Time-calibrated phylogeny adapted from Jones et al.** [43] **with divergence times given for each node.** Dating was calibrated based on the timing of the final uplift of the Trans-Mexican Volcanic Belt (see methods). Taxa with too few occurrence records for niche modeling analyses were trimmed from the phylogeny. (PDF 66 kb) Additional file 2:
**Niche overlap for all pairwise comparisons within the genus** ***Xiphophorus*** **.** Values of Schoener’s *D* are above the diagonal and Warren’s *I* below the diagonal. Outlined are comparisons within each of the major clades for which background similarity and niche equivalency tests were conducted. Species pairs with environmental backgrounds more similar than expected by chance in both directions are indicated by a superscript S. Comparisons that were significant in one direction are denoted by an asterisk (^*^). Only 2 of 64 comparisons had equivalent niches and are denoted by superscript E. Overlap and background similarity were generally highest within the northern swordtails, followed by the southern swordtails, and then the platyfishes. (XLSX 22 kb) Additional file 3:
**Predicted niche occupancy together with the time-calibrated phylogeny were used to reconstruct ancestral tolerances to depict the evolution of the niche for each environmental variable.** Patterns of niche evolution varied among traits and species with some showing a high degree of conservatism within the major clades of the genus and other variables for which divergent clades had considerable overlap in ancestral tolerances. (PDF 296 kb) Additional file 4:
**Relative disparity plots for all twelve environmental variables.** The dashed line indicates disparity under an Brownian model of unconstrained evolution. The solid line shows observed disparity. Values below the unconstrained model are indicative of accumulation of disparity (conservatism) within more inclusive clades (divergence among major clades). Positive values indicate increasing disparity (divergence) within sub-clades. (PDF 69 kb)
