# Supplementary figures and images for: Ecological divergence and conservatism: spatiotemporal patterns of niche evolution in a genus of livebearing fishes (Poeciliidae: Xiphophorus)
Source: BMC Evol Biol. 2016 Feb 19;16:44. doi: 10.1186/s12862-016-0593-4 (PMC4761163; doi:10.1186/s12862-016-0593-4)

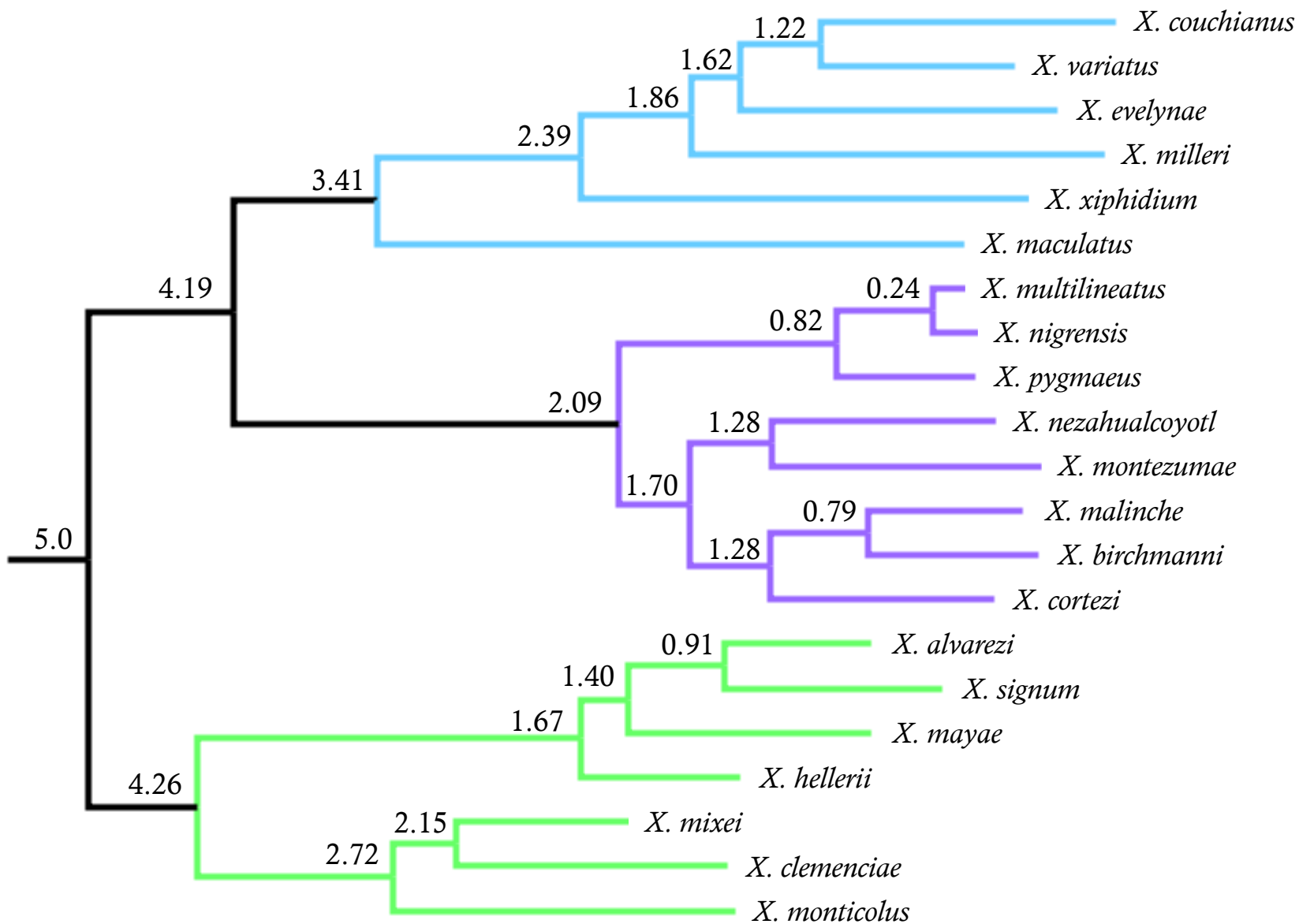

Platyfishes

Northern Swordtails

Southern Swordtails

1 MY

Supplement: Additional file 1: — Time-calibrated phylogeny adapted from Jones et al. [43] with divergence times given for each node. Dating was calibrated based on the timing of the final uplift of the Trans-Mexican Volcanic Belt (see methods). Taxa with too few occurrence records for niche modeling analyses were trimmed from the phylogeny. (PDF 66 kb) [file 12862_2016_593_MOESM1_ESM.pdf]

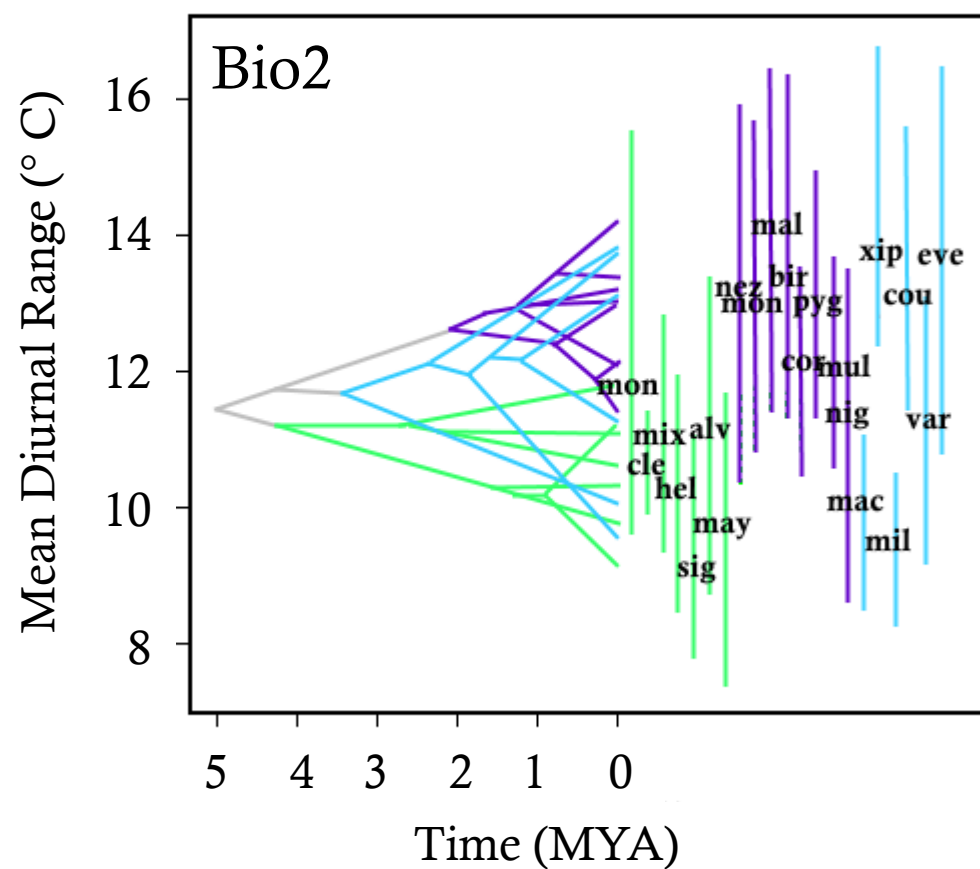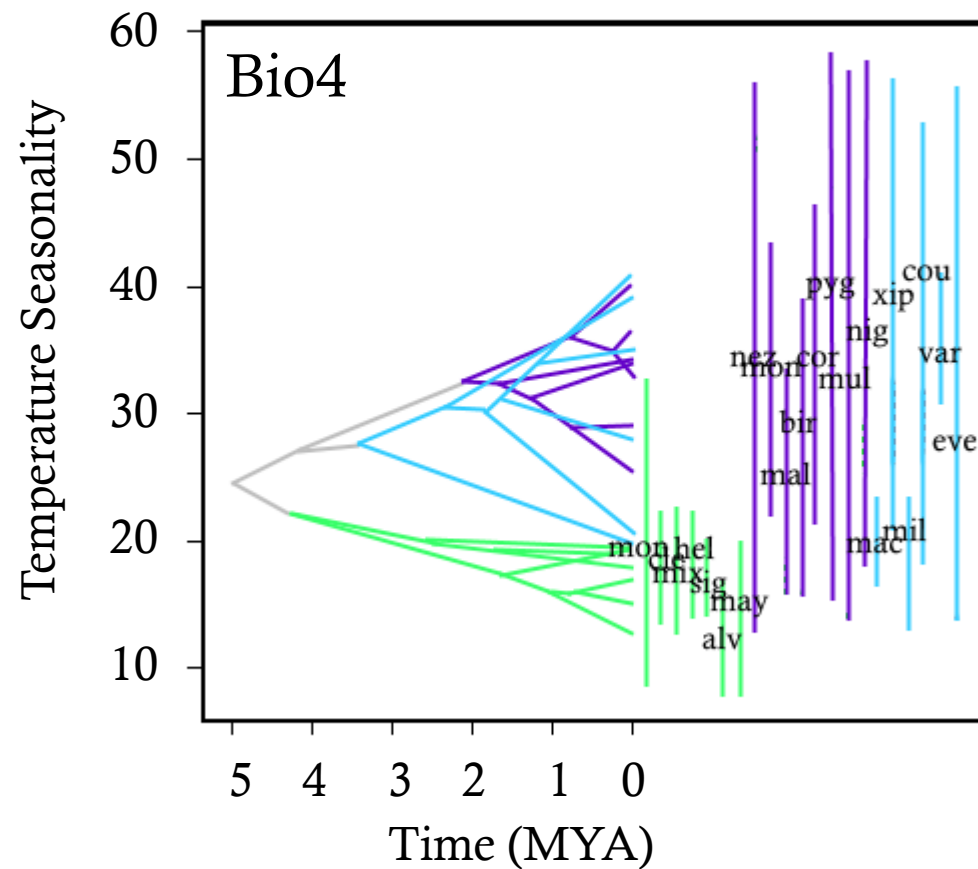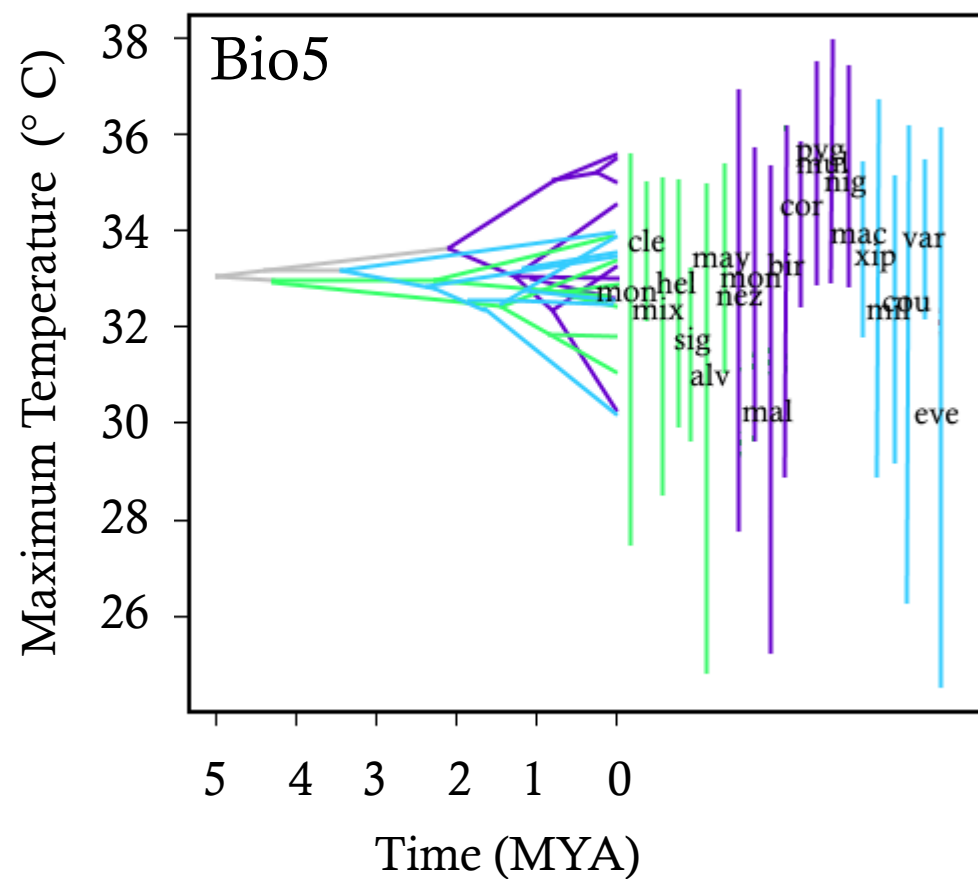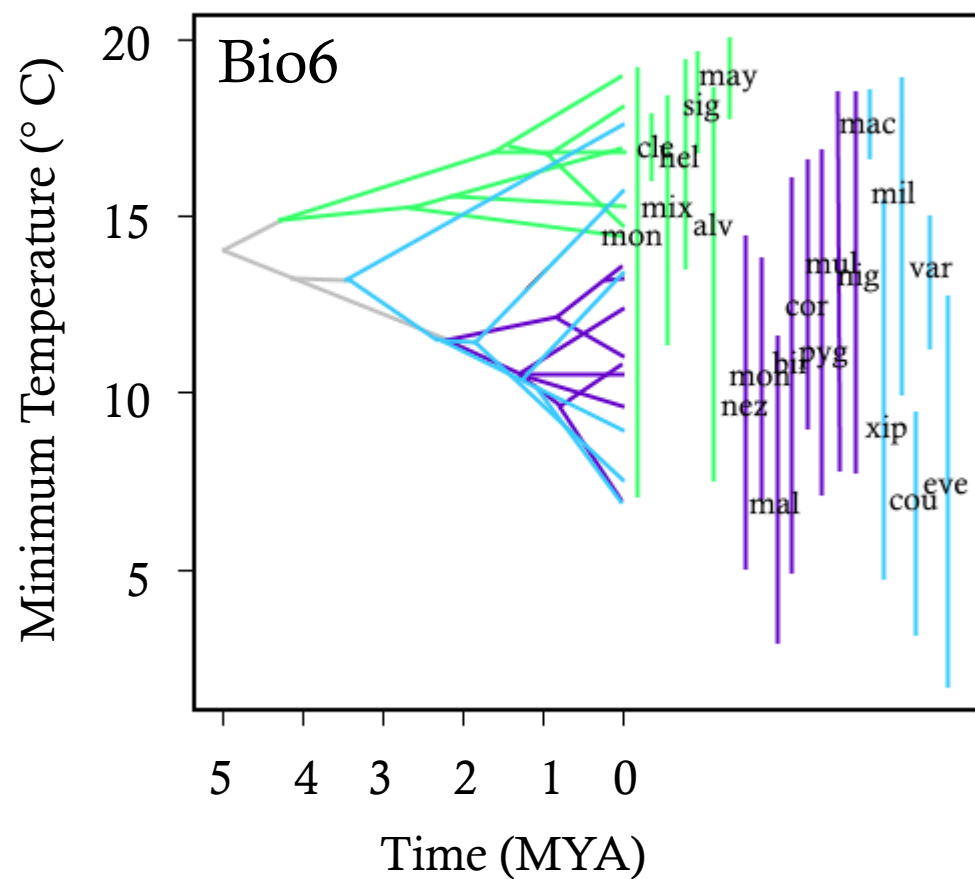

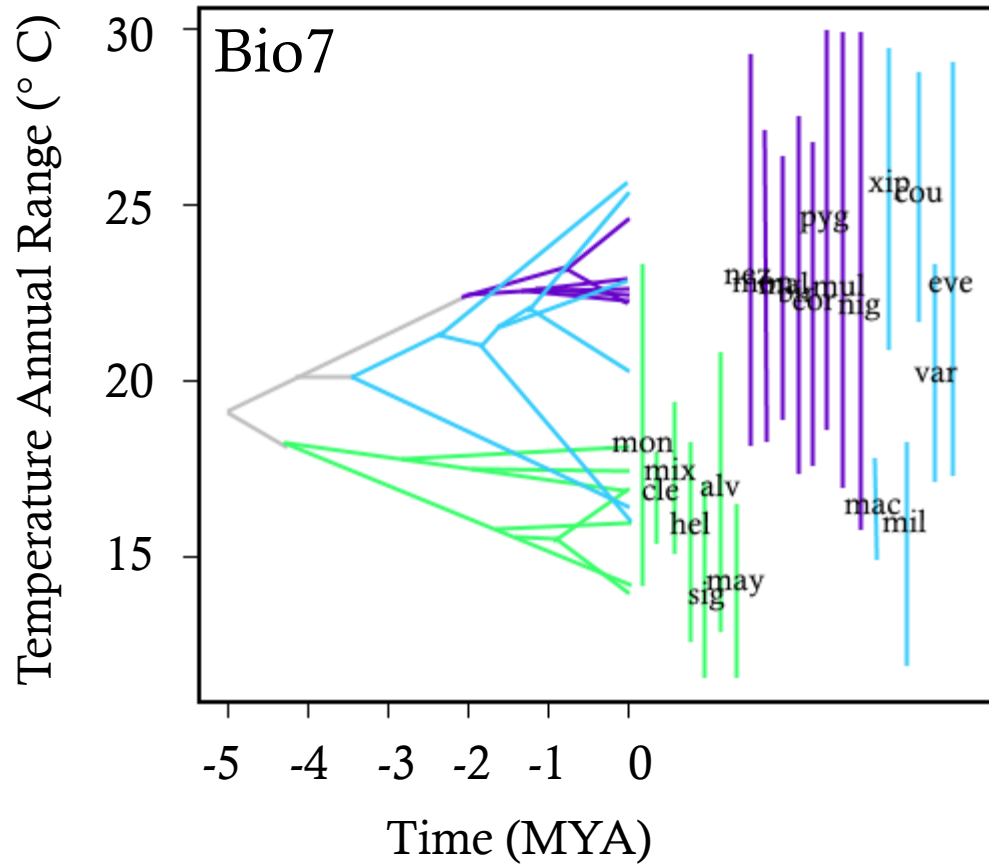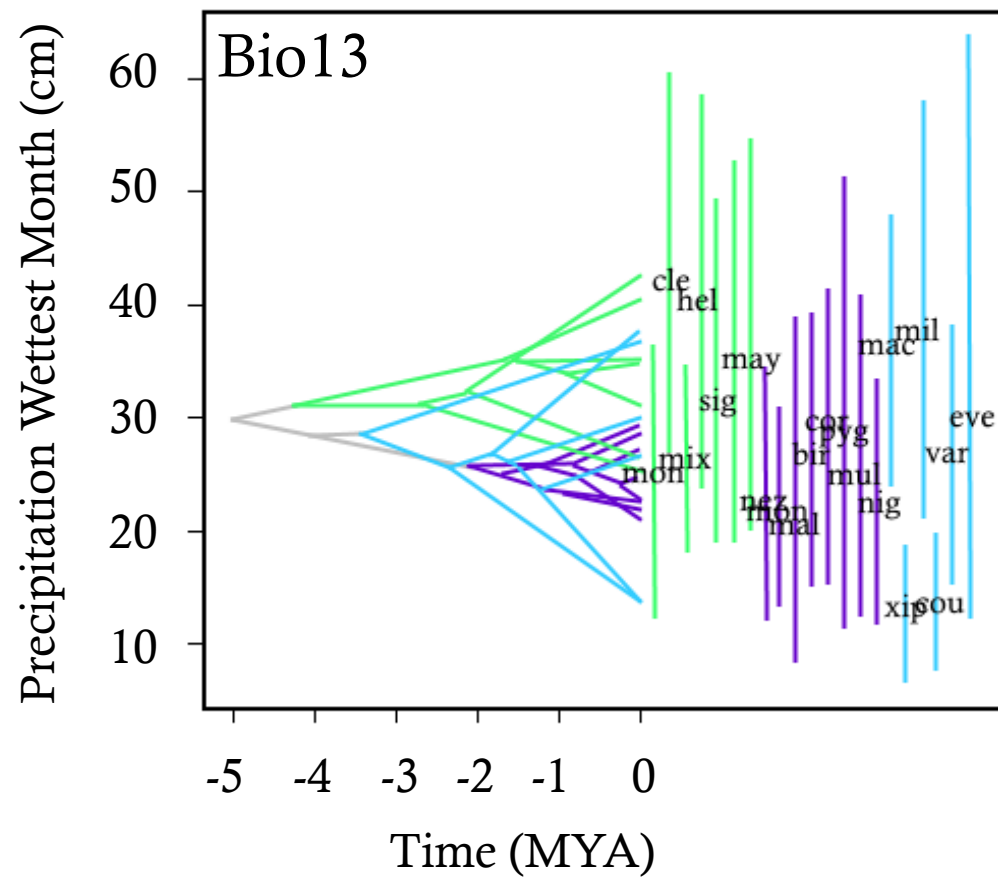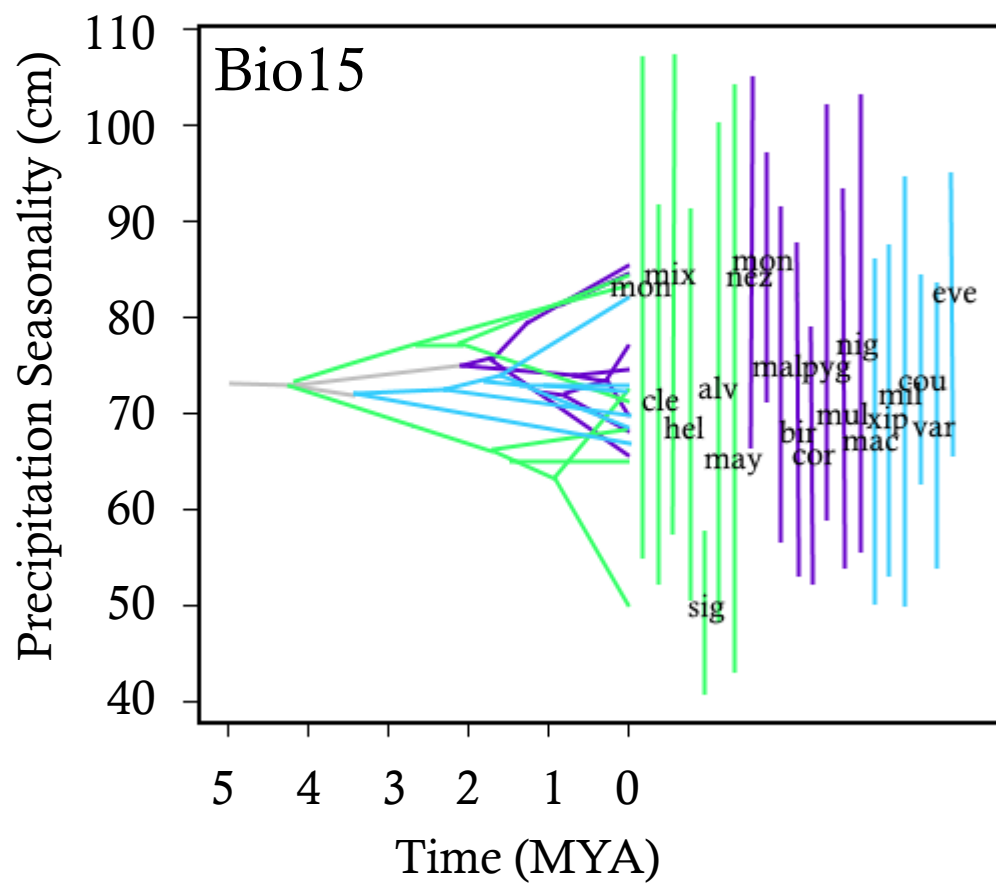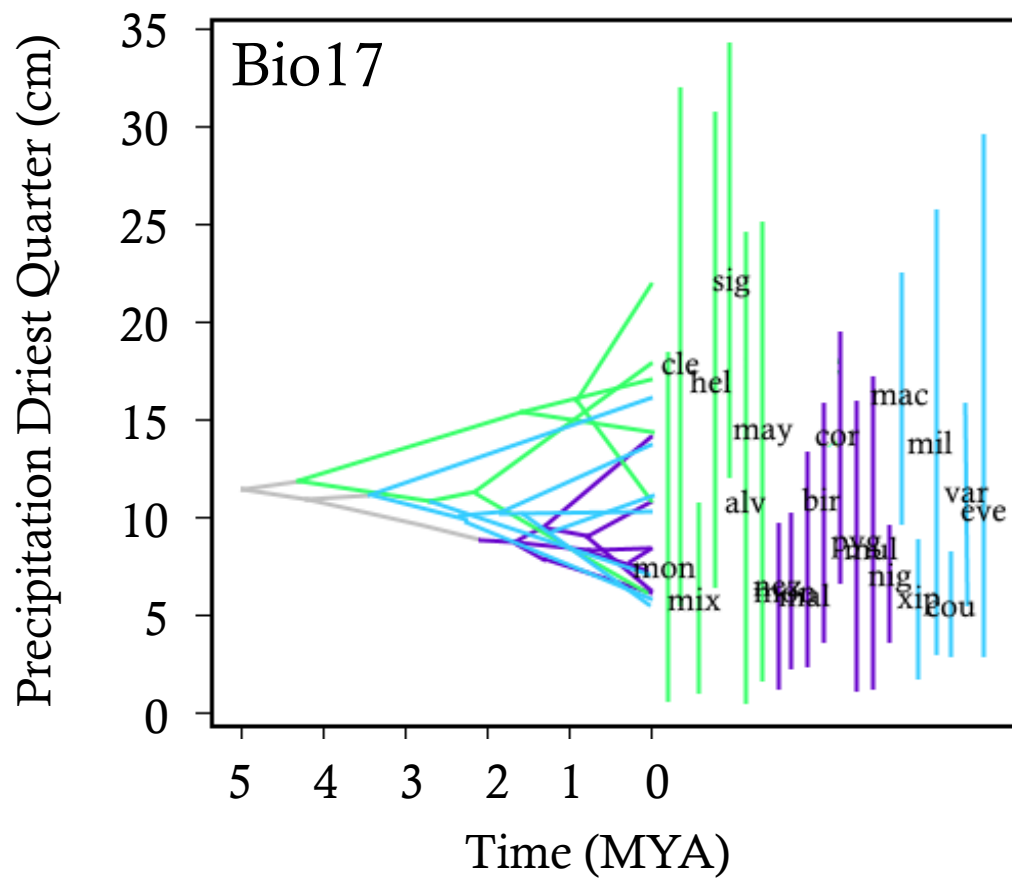

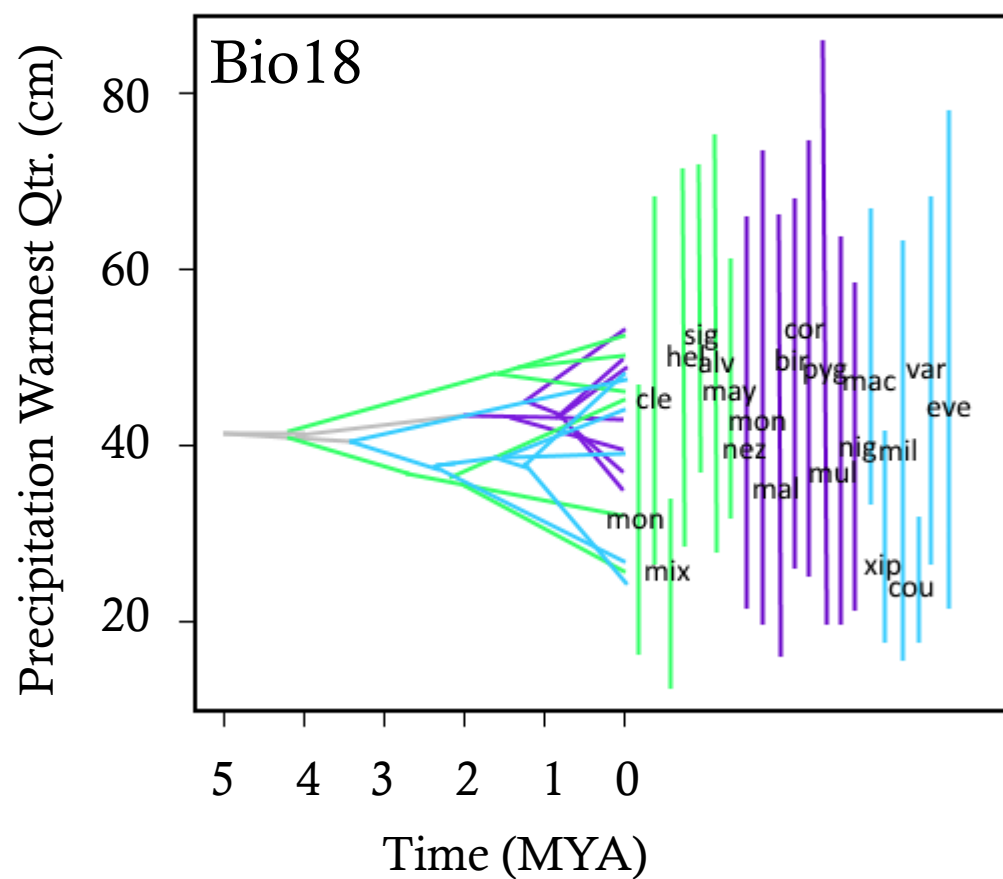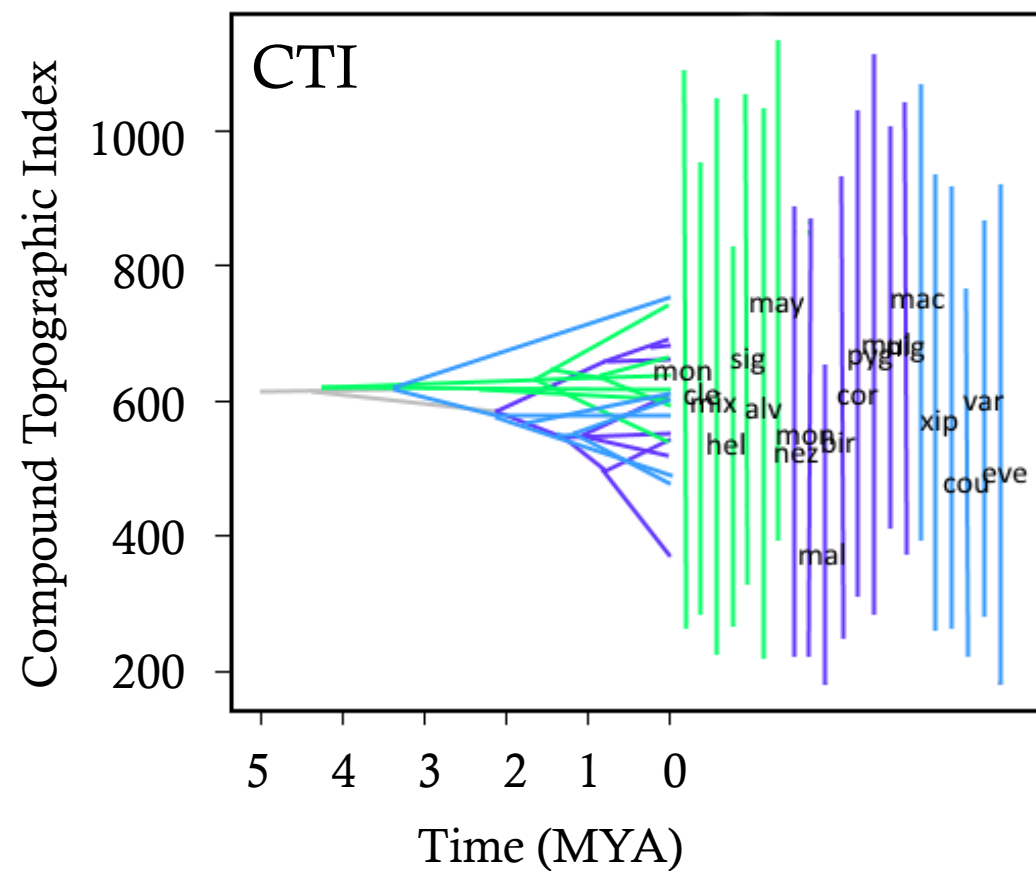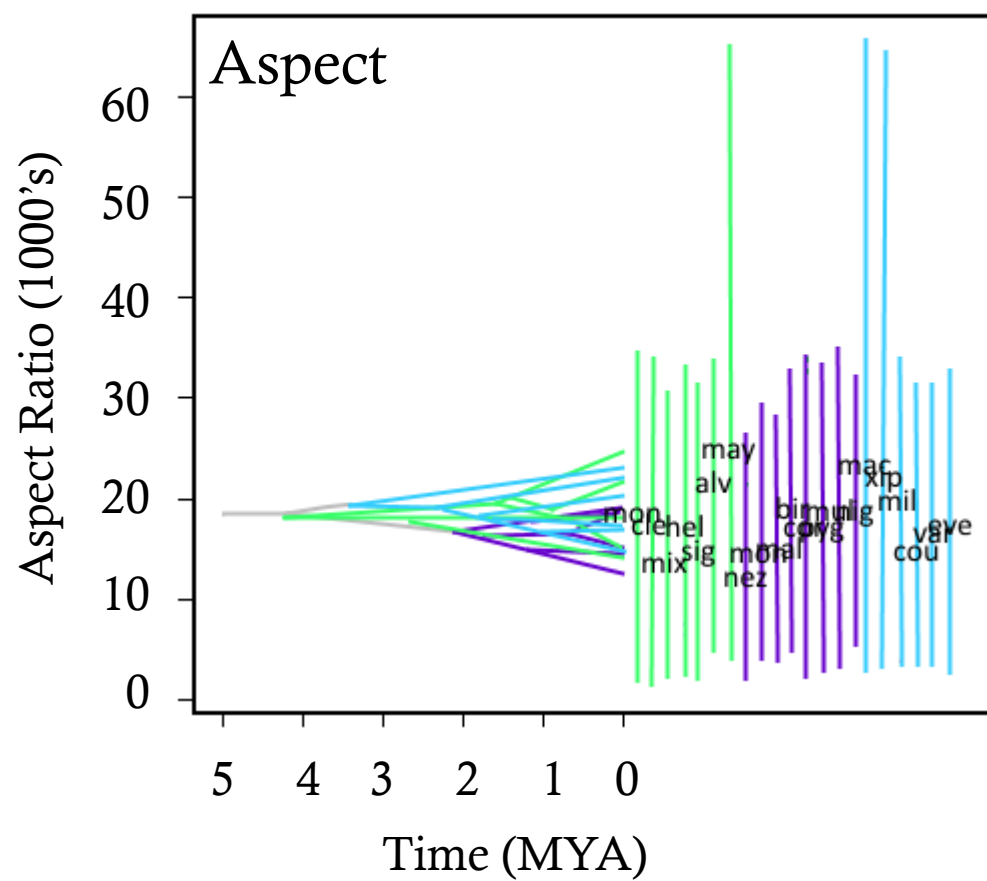

Supplement: Additional file 3: — Predicted niche occupancy together with the time-calibrated phylogeny were used to reconstruct ancestral tolerances to depict the evolution of the niche for each environmental variable. Patterns of niche evolution varied among traits and species with some showing a high degree of conservatism within the major clades of the genus and other variables for which divergent clades had considerable overlap in ancestral tolerances. (PDF 296 kb) [file 12862_2016_593_MOESM3_ESM.pdf]

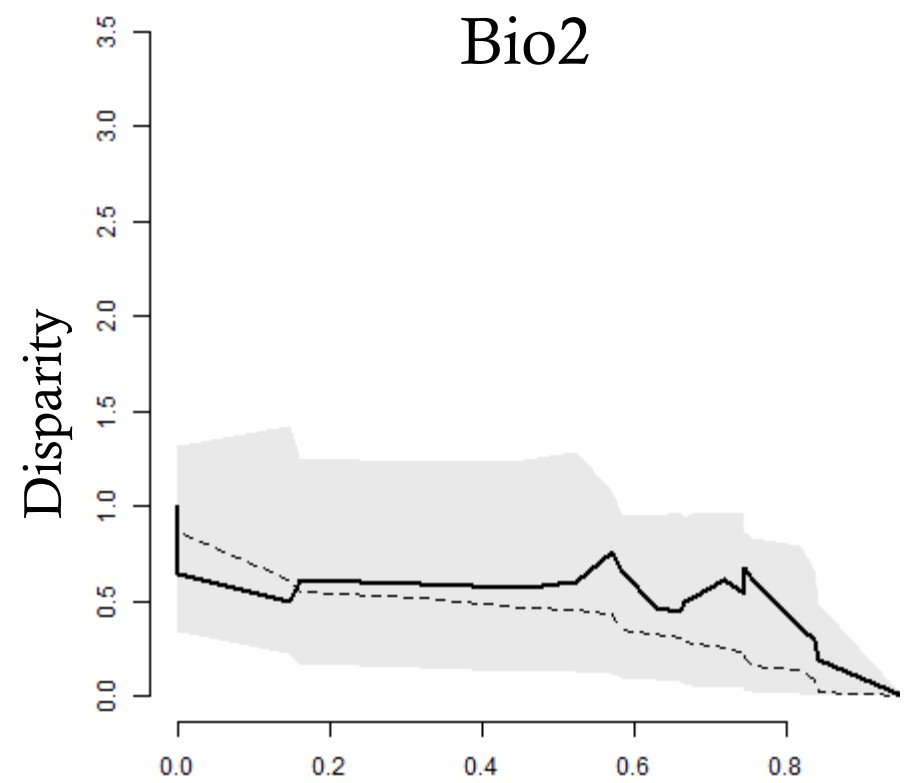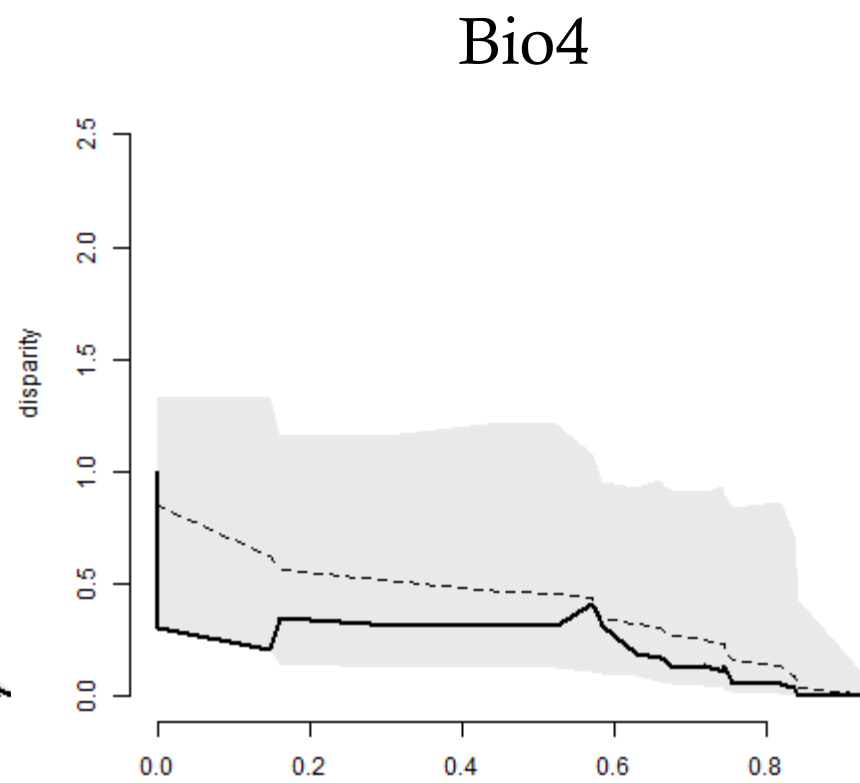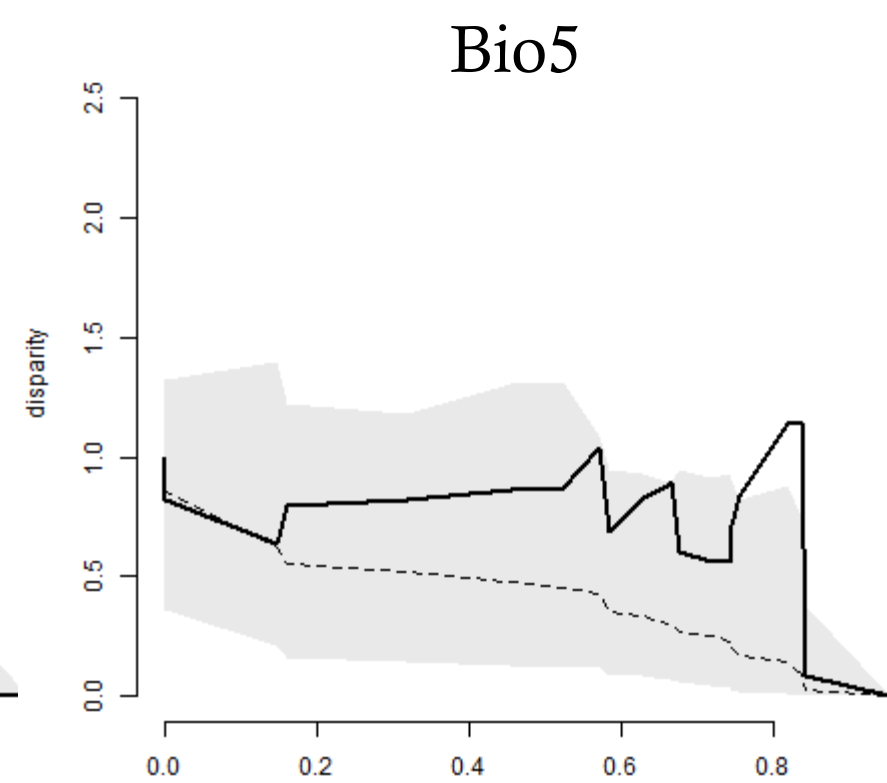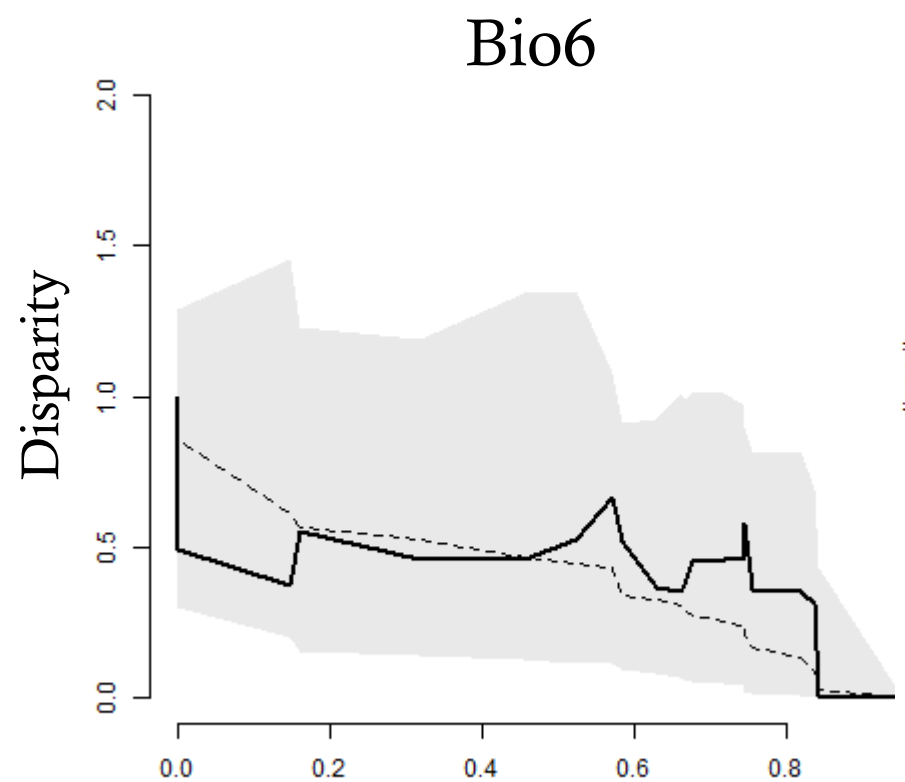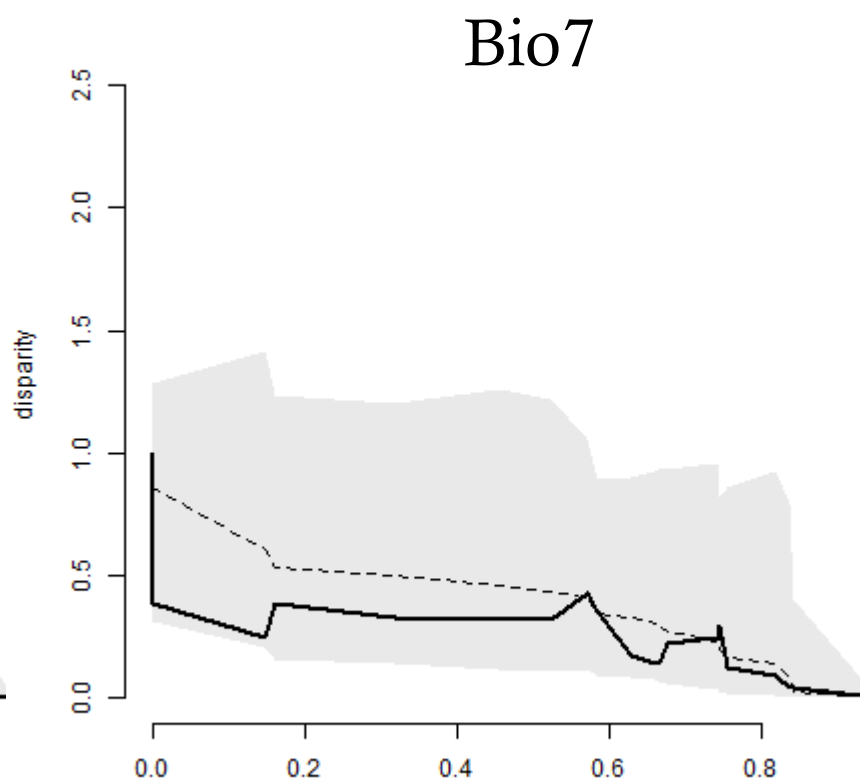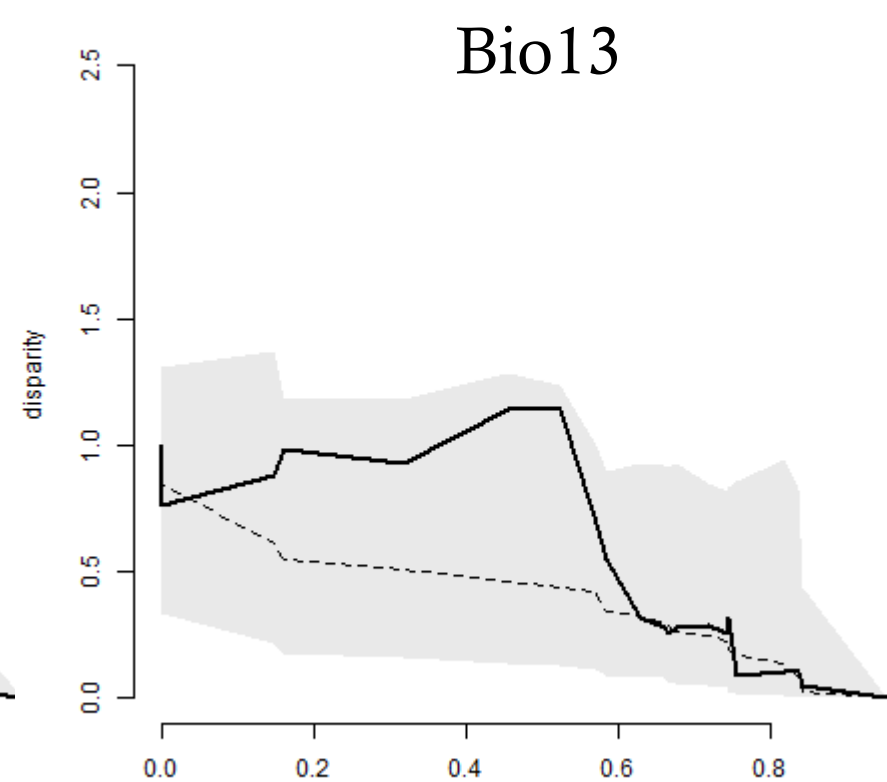

Relative Time

Bio15

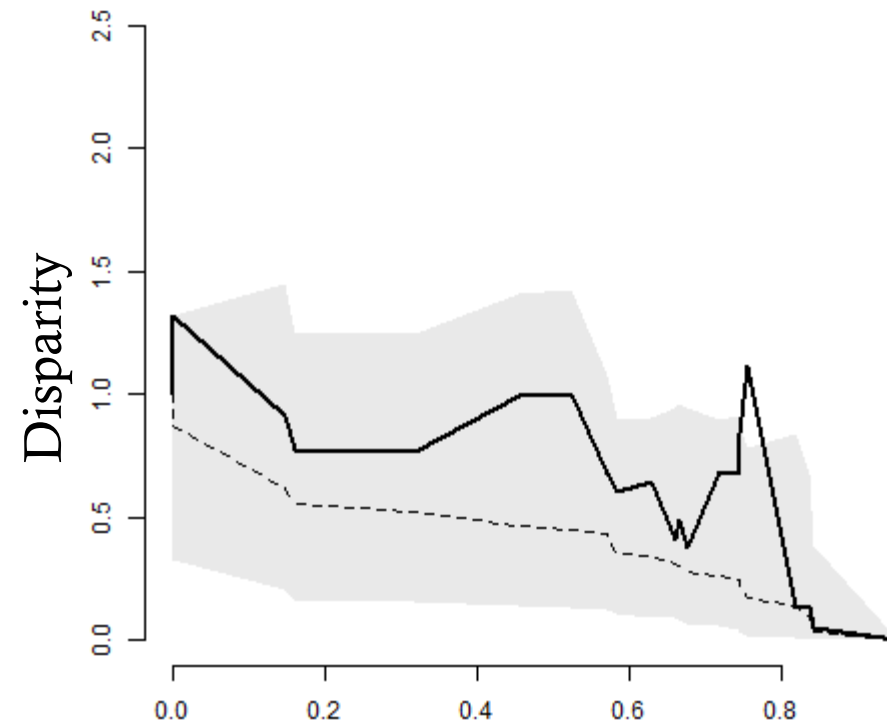

Bio17

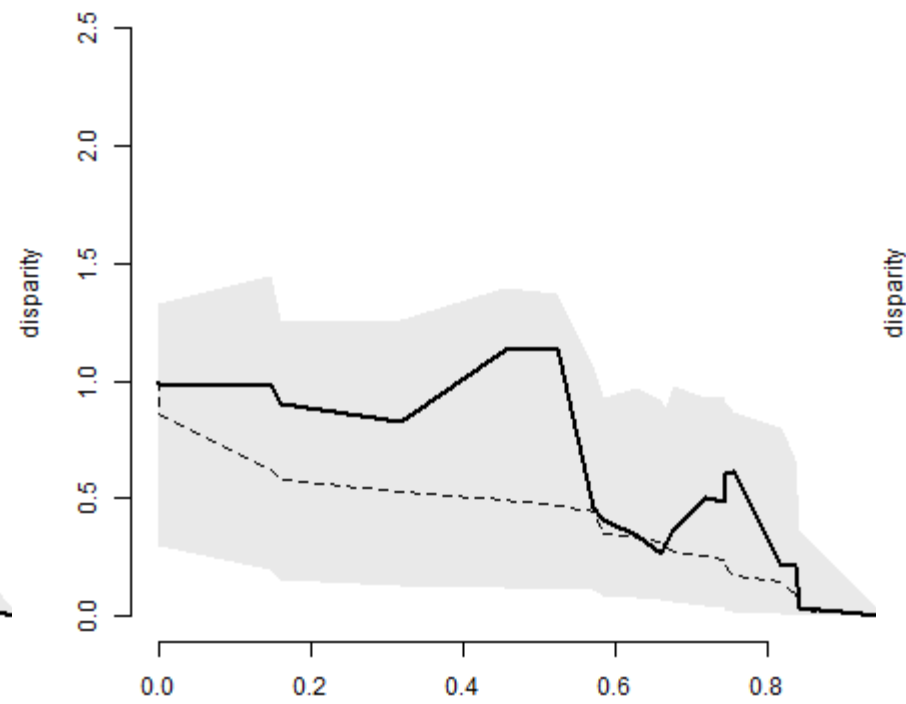

Bio18

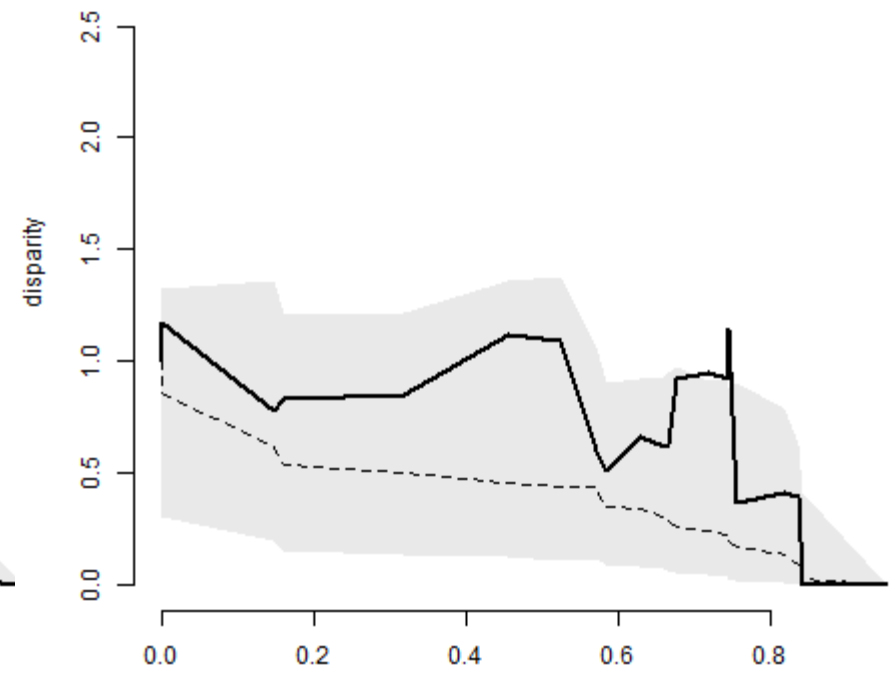

Relative Time

CTI

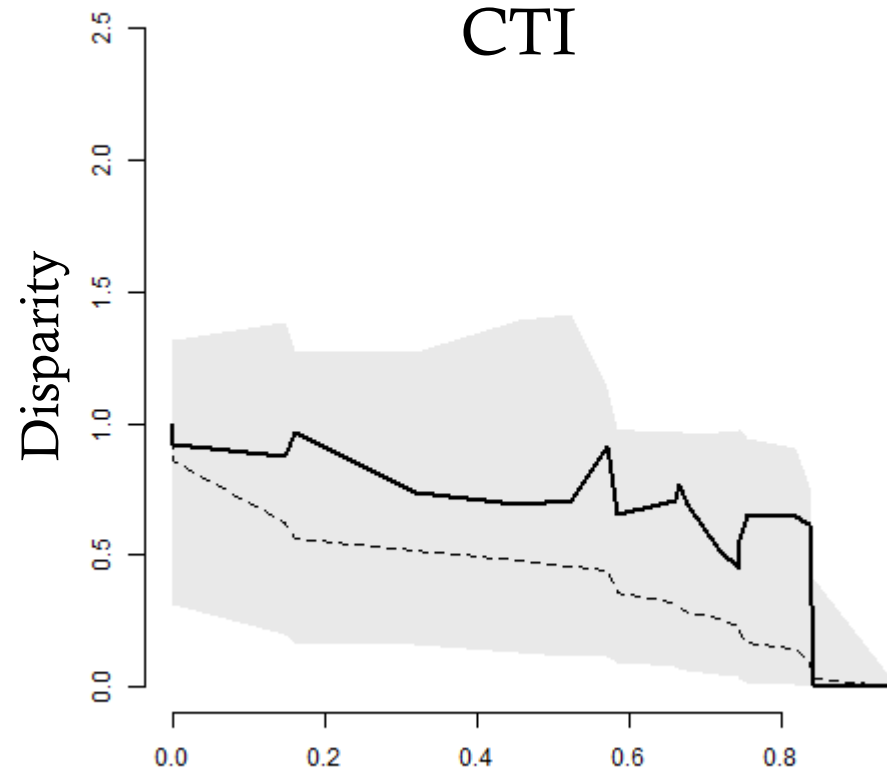

Aspect

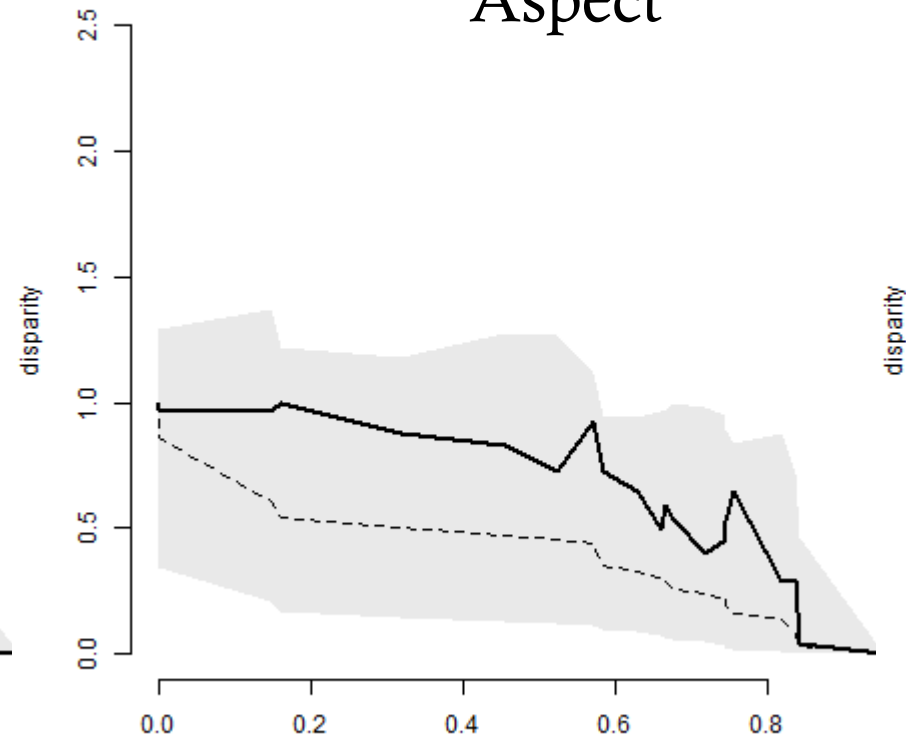

FA

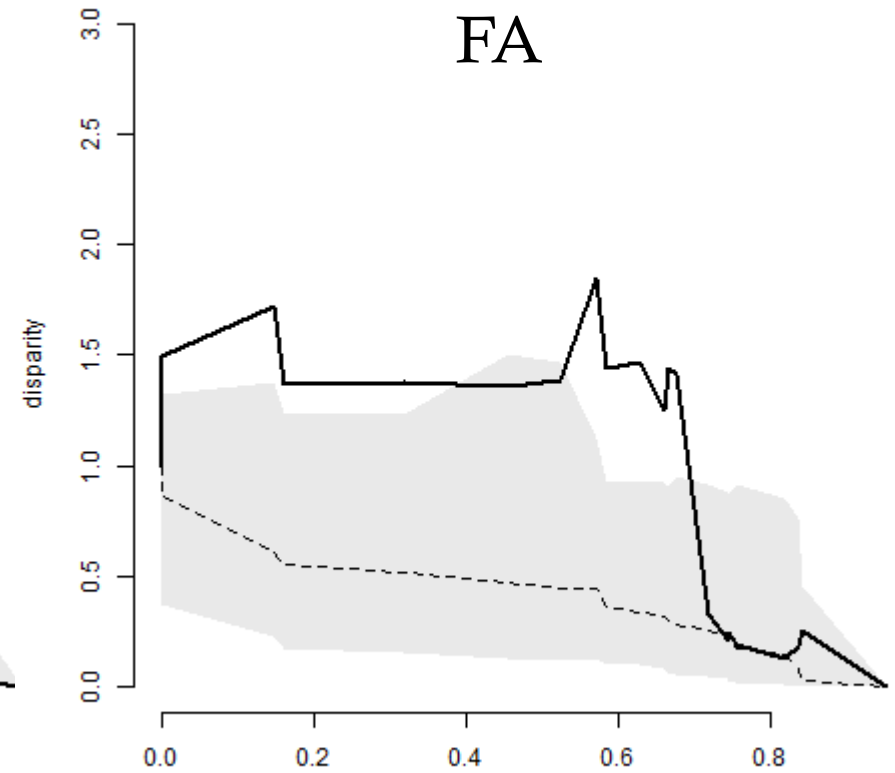

Relative Time

Supplement: Additional file 4: — Relative disparity plots for all twelve environmental variables. The dashed line indicates disparity under an Brownian model of unconstrained evolution. The solid line shows observed disparity. Values below the unconstrained model are indicative of accumulation of disparity (conservatism) within more inclusive clades (divergence among major clades). Positive values indicate increasing disparity (divergence) within sub-clades. (PDF 69 kb) [file 12862_2016_593_MOESM4_ESM.pdf]
